# The origin and biogeographic diversification of fishes in the family Poeciliidae

**DOI:** 10.1371/journal.pone.0172546

**Published:** 2017-03-09

**Authors:** David N. Reznick, Andrew I. Furness, Robert W. Meredith, Mark S. Springer

**Affiliations:** 1 Department of Biology, University of California Riverside, Riverside, California, United States of America; 2 Department of Ecology and Evolutionary Biology, University of California Irvine, Irvine, California, United States of America; 3 Department of Biology and Molecular Biology, Montclair State University, Montclair, New Jersey, United States of America; University of Konstanz, GERMANY

## Abstract

The fish subfamily Poeciliinae (sensu Parenti, 1981) is widely distributed across the Western Hemisphere and a dominant component of the fish communities of Central America. Poeciliids have figured prominently in previous studies on the roles of dispersal and vicariance in shaping current geographic distributions. Most recently, Hrbek et al. combined a DNA-based phylogeny of the family with geological models to provide a biogeographic perspective that emphasized the role of both vicariance and dispersal. Here we expand on that effort with a database enlarged in the quantity of sequence represented per species, in the number of species included, and in an enlarged and more balanced representation of the order Cyprinodontiformes. We combine a robust timetree based upon multiple fossil calibrations with enhanced biogeographic analyses that include ancestral area reconstructions to provide a detailed biogeographic history of this clade. Key features of our results are that the family originated in South America, but its major diversification dates to a later colonization of Central America. We also resolve additional colonizations among South, Central and North America and the Caribbean and consider how this reconstruction contributes to our understanding of the mechanisms of dispersal.

## Introduction

Species in the fish subfamily Poeciliinae (sensu [1]) are prominent features of fresh- and brackish water environments from Argentina in the south to New Jersey and Illinois in the north and on many islands throughout the Caribbean. The subfamily contains approximately 275 species in 27 genera [2, 3]. Members of this subfamily have played important roles in scientific endeavors that range from cancer biology [4] to diverse facets of ecology and evolutionary biology [5]. Their value as research organisms is a function of their short generation times, hardiness in a laboratory environment, and their remarkable biological diversity. One noteworthy feature of the subfamily is variation in maternal provisioning, which ranges from egg laying in one species (*Tomeurus gracilis*) to livebearing with little or no post-fertilization maternal provisioning to having the functional equivalent of a mammalian placenta [6–8]. Other forms of variation that are foci of much of the research effort devoted to this subfamily are traits associated with sexual selection and conflict. The males of some species have highly elaborate ornamentation, including swords, enlarged dorsal fins, laterally compressed bodies and sexual dichromatism associated with elaborate courtship and male-male aggression [9]. Mapping these features on the phylogenetic tree of the subfamily shows that they have all evolved multiple times on different branches of the tree. This presence of multiple, independent origins of biologically significant traits makes the unravelling of the evolutionary history of this subfamily a goal of particular interest because that history can help define the circumstances that promoted the evolution of this diversity.

Rosen and Bailey [10] proposed that the Poeciliinae (sensu [1, 11] originated in Central America because Central America harbors the greatest biodiversity. An alternative hypothesis is that the subfamily originated in South America [12]. This subfamily figured prominently in the development of vicariance biogeography, where parallel patterns of distribution were noted for some poeciliids, reptiles and plants [13, 14]. This parallelism was interpreted as a common response to tectonic activity that successively fractured and rearranged their distributions, creating common patterns of geographic isolation and common pathways for dispersal. The advent of molecular phylogenetics has made it possible to add the specificity gained by integrating well-resolved family trees with estimates of the timing of diversification events and geological events to reconstruct evolutionary history in a more formal fashion. This integration has been applied to individual genera, subgenera or species (e.g. *Brachyraphis* [15]- *Gambusia*–[16, 17]; *Poeciliopsis*—[18, 19]; *Girardinu*s—[20]; *Pseudoxiphophorus*–[21]; *Poecilia*–[22, 23]; *Limia*–[24]; *Mollienesia*–[25]). Hrbek et al. [12] presented the most recent comprehensive family-wide analysis.

Hrbek et al. [12] resolved the debate about the origins and history of dispersal of this group into three models, then deployed molecular phylogenetics to discriminate among them. One model was that the subfamily originated during the Cretaceous and had an early distribution that spanned South, Central and North America and hence that there was an earlier land bridge that joined the continents. A second alternative was that the subfamily originated in Central America after the split of North and South America and did not colonize South American until after the formation of the Panamanian land bridge. A third alternative was again that the subfamily originated during the Cretaceous, had an early distribution from Middle to South America, colonized the Greater Antilles from South America during the Eocene, then dispersed in both directions between South and Central America after the closure of the Isthmus of Panama.

Hrbek et al.[12] concluded that the Poeciliinae originated in South America during the Cretaceous. There results suggest a connection between Central and South America during the Cretaceous followed by bidirectional dispersal between the two regions, early colonization of the Greater Antilles, then independent radiations within all three regions. They argue that the colonization of Middle and North America was during the Eocene-Oligocene transition and was most likely from the Greater Antilles. Here we revisit Hrbek et al.’s [12] analysis with the benefit of: 1) the enhancement of the data on which the analysis is based, both in terms of the number and representation of species and in the number of loci per species, 2) a time tree based upon 16 primary fossil calibrations, and 3) the application of analytical techniques that enabled us to reconstruct formal ancestral area reconstructions and hence make inferences about the direction and timing of major dispersion events and subsequent radiations.

## Materials and methods

The UCR IACUC committee approved of the research contained in this manuscript. All of this research was conducted under the auspices of an Animal Use Protocol administered by the IACUC at U. C. Riverside. UCR has Animal Welfare Assurance #A3439-01. The associated animal use protocol number is A-20140003.

### Timetree analyses

We performed molecular dating analyses for the subfamily Poeciliinae by integrating the phylogeny presented by Pollux et al. [9] with 16 primary fossil calibration points. We estimated divergence times for Pollux et al.’s [9] RAxML tree and data set, which includes 293 Cyprinodontiformes and outgroups. We used the mcmctree program in PAML 4.4c [26] with (1) autocorrelated rates and hard-bounded constraints and (2) independent rates and hard-bounded constraints. A complete summary of both time trees is presented in newick format as S1 Table. Mcmctree implements Rannala and Yang’s [27] MCMC algorithms. We used six data partitions, each of which was allowed to have its own GTR + Γ model of sequence evolution. We employed a small number of partitions (6) to make mcmctree computationally feasible. Partitions were designed to maximize taxonomic overlap among genes in each partition and were as follows: six loci (*TBR1*, *PTR*, *ZIC1*, *PLAG12*, *RYR3*, *SRBE*) from Li et al. [28], six loci (*CCND1*, *D2*, *D8*, *D29*, *RAB27*, *T36*) with strong taxonomic sampling from *Xiphophorus*, β-ACTIN sequences for *Girardinus* spp. that have minimal taxonomic overlap with other studies, seven nuclear genes (*GLYT*, *X-SRC*, *ENC1*, *Rag1*, *RH*, *SH3PX3*, *MYH6*) with extensive taxonomic representation in Pollux et al. [9], mitochondrial tRNAs and rRNAs, and mitochondrial protein-coding genes (*CYTB*, *ND1*, *ND2*, *COI*). We set one time unit = 100 million years. Analyses were performed with cleandata = 0. Shape (α) and scale (β) parameters for the gamma prior of the overall rate parameter μ (i.e., rgene_gamma in mcmctree) were 1 and 2.11, respectively. Calculations for the shape and scale parameters of the gamma prior for the rate-drift parameter assumed an age of 157.5 million years for the most recent common ancestor of Otocephala. We employed minimum and maximum constraints at 16 nodes including constraints at ten nodes from Furness et al. [29]. Minimum ages were based on the oldest crown fossils that are assignable to each clade. Maximum ages were based on stratigraphic bounding, phylogenetic bracketing, and phylogenetic uncertainty [29–32]. Chains were run for 100,000 generations after a burn-in of 10,000 generations, and were sampled every 20 generations.

Minimum and maximum constraints on 16 calibrated nodes were as follows:

1. Tetraodontidae. **Minimum = 32.25 Ma; maximum = 56.0 Ma.** The geologically oldest representative of crown Tetraodontidae is *A*. *winterbottomi*, which dates to the early Rupelian (34–32 Ma)[33]. The maximum age for Tetraodontidae allows for the possibility that the fossil genus *Eotetraodon* is one of the first two outgroups to crown Tetraodontidae [34, 35]. See Furness et al. [29] for a detailed discussion of minimum and maximum calibrations for this node. Betancur-R et al. [36] used similar minimum and maximum ages of 32.0 and 50.0 Ma, respectively.
2. Tetraodontiformes + Lophiiformes. **Minimum = 96.9 Ma; maximum = 126.0 Ma.** We used minimum and maximum dates from Furness et al. [29] for this node. Tetraodontiformed and Lophiiformes are sister taxa in Miya et al. [37]. The oldest fossils belonging to Lophiiformes (e.g., anglerfish, frogfish) include frogfish (i.e., *Eophryne barbutii*) and batfish (i.e., *Tarkus*) from early Eocene (Ypresian) deposits in Italy [38, 39]. The oldest stem Tetraodontiformes fossil is *Plectocretacicus clarae* from the Cenomanian of Lebanon [40]. *P*. *clarae* has a minimum age of 96.9 Ma [41]. The maximum age is based on stratigraphic bounding and is 126.0 Ma.
3. Moronidae + Lutjanidae. **Minimum = 47 Ma; maximum = 84.2 Ma.** We used minimum and maximum dates from Furness et al. [29]. The minimum age is based on fossil moronids from middle Eocene Messel deposits that are ~ 47 Ma [42]. Skeletal remains of moronids are also known from Lutetian deposits of the Green River Formation, Wyoming (e.g. *Priscacara serrate*, [43]). Lutjanid skeletal remains are known from Lutetian deposits of the Monte Bolca locality (e.g. *Ottaviania leptacanthus*, [44]). The maximum age for this node (83.5 +/- 0.7 = 84.2 Ma) is based on otoliths from the Late Campanian with putative moronid affinities [45]. Alfaro et al. [46] and Santini et al. [47] both used a minimum of 74 Ma for crown Moronidae base on fish otoliths (*Morone* sp.) from the Late Cretaceous Coffee Sand of Mississippi [48], but in a later publication Nolf [49] refers to the oldest putative moronid fossil from the Cretaceous as? Monoridae indet. We therefore used a younger minimum age as in Furness et al. [29].
4. Zoarcoidea + Gasterosteiformes. **Minimum = 13.0 Ma; maximum = 84.2 Ma.** Minimum and maximum ages are from Furness et al. [29]. The minimum age of 13.0 Ma is established by *Gasterosteus aculeatus*, which is known from the Monterey Formation, California (13–13.3 My; Serravallian; [50]) and is the oldest known non-indostomid gasterosteiform. The maximum age allows for the possibility that the Campanian *Gasterorhamphosus* has stem group affinities with Zoarcoidea + Gasterosteiformes.
5. Poeciliinae + Anablepidae. **Minimum = 39.9 Ma; maximum = 71.2 Ma.** Minimum and maximum ages are from Furness et al. [29]. The minimum age is based on undescribed poeciliid fossils that are known from the Lumbrera Formation in Argentina [51]. As noted by Furness et al. [29], geological data suggest an upper Ypresian-lower Lutetian age for the Faja Verde of the Lumbrera Formation. However, the minimum age in only 39.9 Ma based on U/Pb zircon dating of a tuff bed that overlies this formation [51]. The maximum is established by cf. Cyprinodontiformes fossils from the El Molino Formation in Bolivia of Maastrichtian to Paleocene age [52] that have uncertain relationships to living taxa. Santini et al. [47] employed a similar calibration (minimum = 55 Ma, maximum = 99 Ma) for Poeciliidae to Fundulidae.
6. Otocephala = Ostarioclupeomorpha (Ostariophysi + Clupeomorpha). **Minimum = 149.85; maximum = 165.2 Ma.** The oldest crown Ostarioclupeomorpha is *Tischlingerichthys viohli* from the upper Tithonian of Muhlheim, Bavaria, Germany [53, 54]. We followed Benton et al. [41] and Furness et al. [29] and employed a minimum age of 149.85 Ma based on *T*. *viohli*. The maximum age is based on stratigraphic bounding as in Furness et al [29]. Alfaro et al. [46] employed minimum and maximum ages of 149 and 152 Ma for this node.
7. Clupeocephala (= Otocephala + Euteleostei). **Minimum = 149.85 Ma; maximum = 165.2 Ma.** The oldest crown otocephalan is *Tischlingerichthys viohli* from the upper Tithonian of Muhlheim, Bavaria, Germany [53, 54]. The oldest euteleosts are *Leptolepides* and *Orthogonikleithrus* from the same deposits [41]. The maximum age is based on stratigraphic bounding. Our minimum and maximum agree with Benton et al. [55]. Subsequently, Benton et al. [55] suggested a much older maximum (235 Ma) for this clade while acknowledging that a maximum bound for crown Clupeocephala can only be defined arbitrarily.
8. Clupeomorpha. **Minimum = 82.8 Ma; maximum = 149.5 Ma.** Clupeomorph taxa in our phylogeny include one clupeid and two pristigasteroids. The minimum is based on *Gasteroclupea branisai*, which is found in Santonian deposits of Bolivia (Chaunaca Formation; [56]) and has been treated as Clupeidae [57], Pristigasteroidea [57], or Pristigasteridae [56]. The oldest stem clupeomorphs are Neocomian (i.e.. *Scutatuspinosus itapagipensis*, [58, 59]. The base of the Neocomian is 149.5 Ma.
9. Siluriformes + Characiformes. **Minimum = 92.8 Ma; maximum = 126.0 Ma.**Bertini et al. [60] described Siluriformes indet. from skeletal elements from the lower portion of Adamantina Formation (Locality 99, Brazil). The age of this formation is Turonian-Santonian [61]. Cione and Prasad [62] suggest the oldest catfish records (cf. Ariidae) are Campanian (see [52]). The oldest fossils assignable to the characiform are isolated teeth from the Wadi Milk Formation (Cenomanian), Sudan [63]. We based the minimum age for this node on these Cenomanian fossils (min = 93.6 +/- 0.8 = 92.8 Ma). The placement of the fossil species *Santanichthys diasii* is controversial. Historically, *Santanichthys* has been treated as Clupeocephala *incertae sedis*, Clupeomorpha, or Clupavidae (reviewed in Malabarba and Malabarba, [63]. Most recently, Filleul and Maisey [64] place *Santanichthys* in Characiformes (Otophysi) while Malabarba and Malabarba [63] and Diogo et al. [65] argue that *Santanichthys* is a stem otophysian. Santini et al. [47] treated *Santanichthys diasii* as the oldest stem characiform. We treat *Santanichthys diasii* as Otophysi *incertae sedis*. Stratigraphic bounding suggests a maximum age of 126.0 Ma.
10. Ostariophysi. **Minimum = 133.9 Ma; maximum = 155.6 Ma.** The oldest undisputed crown ostariophysian is the gonorynchiform *Rubiesichthys gregalis* (Valanginian-Berriasian, Serra del Montsec, Lerida Province, Spain) [66]. The minimum age for Ostariophysi is based on the upper boundary of the Valanginian (133.9 Ma). The maximum age based on phylogenetic bracketing/phylogenetic uncertainty is based on *Tischlingerichthys viohli* (Tithonian, Solnhofen Limestone Formation, Mühlhei, Bavaria, Germany; [53, 54]), which has been traditionally treated as a stem characiform [54], but more recently [65] has been hypothesized as a stem ostariophysian. In this study we treat *Tischlingerichthys viohli* as Ostariophysi *incertae sedis*. The maximum age based on stratigraphic bounding is two chronologic units below the Berriasian, i.e., 155.6 Ma.
11. Esociformes + Salmoniformes. **Minimum = 70.0 Ma; maximum = 126.0 Ma.** We used minimum and maximum dates from Furness et al. [29] for Esociformes + Salmoniformes. *Estesesox foxi* is the oldest esociform fossil and is from the Aquilan (early Campanian), Milk River Formation, Alberta [67, 68]. We use the end of the Campanian (70.6 + 0.6 = 70.0 Ma) to establish a minimum age for this clade. The maximum age is based on *Helgolandichthys schmidi* (early Aptian, Tock, Helgolang, Germany), which was originally described as a salmoniform [69] although we are unaware of any phylogenetic studies that have included this taxon.
12. Macrouridae to Gadidae (in Gadiformes). **Minimum = 58.5 Ma; maximum = 131.5 Ma.** Minimum and maximum dates are from Furness et al. [29]. The oldest crown taxa in this clade are macrourids and gadids that are known from otoliths of Selandian age [70, 71]. The definitive sister-taxon to Gadiformes is unclear, but the candidate lineages include other Neoteleostei, among which are Barremian Aulopiformes [72] that establish a maximum age of 131.5 Ma.
13. Cichlidae. **Minimum = 40.2 Ma; maximum = 100.5 Ma.** Our minimum and maximum dates are from Furness et al.[29]. The oldest crown cichlids are New World fossils (e.g. *Proterocara argentina*, *Gymnogeophagus eocenicus*) from the Lumbrera Formation of Argentina and are Ypresian-Lower Lutetian (Eocene) in age [73]. Phylogenetic analyses (e.g., [74]) recover *Proterocara argentina* and *Gymnogeophagus eocenicus* as crown members of the South American Cichlinae and establish a minimum age for Cichlidae [29]. The maximum age allows for phylogenetic uncertainty in the placement of the *Plectocretacicus clarae* from the Cenomanian, i.e., 99.6 +/- 0.9 = 100.5 Ma.
14. Neoteleostei. **Minimum = 124 Ma; maximum = 154.8 Ma.** Minimum and maximum dates are from Furness et al.[29]. The oldest neoteleostians (e.g. *Atolvorator longipectoralis*) belong to Aulopiformes and are Barremian in age [72]. These fossils establish a minimum age of 124 Ma for Neoteleostei. Pachyrhizodontoidea are stem euteleosts [75] or clupeocephalans *incertae sedis* [76] and may belong to the second outgroup to Neoteleostei. The oldest pachyrhizodontoid is an indeterminant Tithonian species from Banos del Flaco Formation, Termas del Flaco, Chile [77], which establishes a maximum age of 154.8 Ma.
15. Goodeidae. **Minimum = 5.332 Ma; maximum = 23.03 Ma.** *Tapatia occidentalis* (Jalisco, Mexico, late Miocene; [78]) and *Empetrichthys erdidi* (Posey Canyon shale, late Miocene, southern California;[79]) are the oldest known goodeids [80].[81, 82]. Given the uncertain position of *Tapatia* within Goodeidae we used this taxon as a minimum for crown Goodeidae. The maximum is based on stratigraphic bounding and is 23.03 Ma.
16. *Empetrichthys* to *Crenichthys*. **Minimum = 5.332 Ma; maximum = 23.03 Ma.** *Empetrichthys erdisi* is known from the Posey Canyon Shale. This member of the Peace Valley Formation was originally considered to be Pliocene in age (e.g., [79]), but more recent evidence [83] suggests a Miocene age. We used the top of the Miocene as the minimum age for the split between *Empetrichthys* and *Crenichthys*, and the base of the Miocene as the maximum based on stratigraphic bounding.

### Biogeographical reconstructions

Ancestral areas were reconstructed with minimum area change (MAC) parsimony [32] and BioGeoBears [84–86] for 252 cyprinodontiform taxa from Pollux et al.’s [9] data matrix (area assignments for each taxa are summarized in S2 Table). We performed the BioGeoBears analysis with the timetree that was reconstructed with mcmctree using autocorrelated rates and hard-bounded constraints. MAC parsimony analyses were performed with Mesquite [87]. Taxa were coded for their occurrence in one or more of the following six areas: North America (excluding Mexico), Central America + Mexico, South America, West Indies, Africa, Europe + Asia (Supplementary Information). We allowed a maximum of three areas per node, which exceeds the range of extant taxa, all of which are confined to one or two areas. Analyses with BioGeoBears were performed in R [85] and employed the dispersal-extinction-cladogenesis (DEC) model of Ree and Smith [88] and the DEC + J model of Matzke [84, 86] that combines DEC with an allowance for jump dispersal. DEC and DEC + J analyses were performed with a maximum size of three areas. DEC and DEC + J models were compared using a likelihood ratio test.

## Results

### Molecular dating analyses

**Fig 1:**
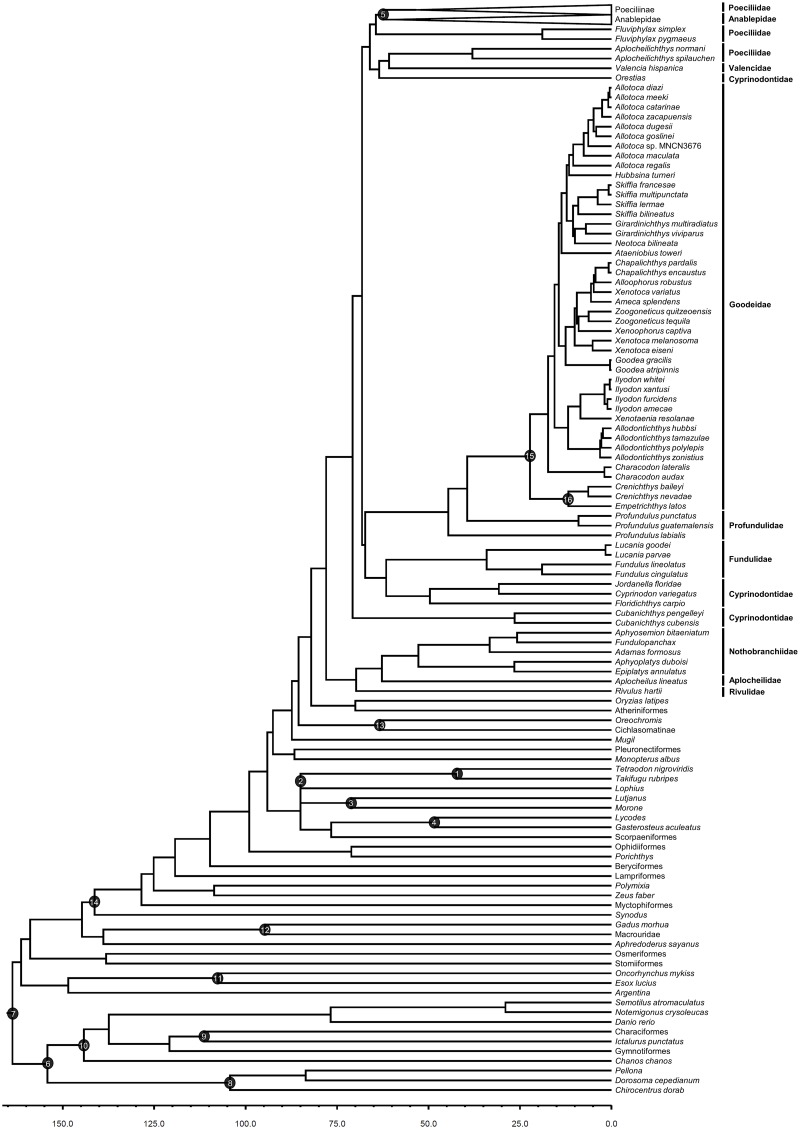
Timetree for 293 Cyprinodontiformes and outgroup taxa. Divergence times were estimated for Pollux et al.’s [9]RAxML tree using the mcmctree program in PAML 4.4c [26] with autocorrelated rates and hard-bounded constraints. Numbered nodes correspond to the sixteen fossil calibrations described in Materials and Methods. The estimated age for Tetraodontiformes to Lophiiformes (85.0 Ma) is younger than the minimum calibration age (96.9 Ma) for this node. Family names (right) are indicated for species in the order Cyprinodontiformes. The clades Poeciliinae and Anablepidae (top) have been collapsed; an expanded view of these clades can be found in Fig 2. The full timetree is available in newick format (S1 Table).

**Fig 2:**
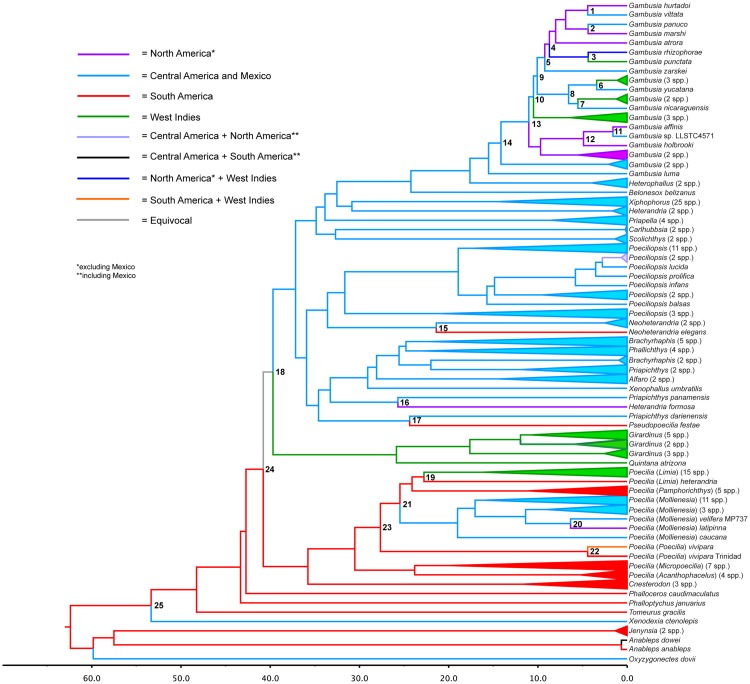
Timetree and ancestral area reconstructions for Poeciliinae + Anablepidae. Divergence times were estimated for Pollux et al.’s [9] RAxML tree using the mcmctree program in PAML 4.4c [26] with autocorrelated rates and hard-bounded constraints. Ancestral areas were reconstructed with minimum area change parsimony [32].

Full timetrees (293 taxa from Pollux et al., [9]) based on autocorrelated and independent rates, including 95% credibility intervals, are provided in Supplementary Information (S1 Table). Fig 1 shows the full tree and the location of the 16 calibrated nodes while Fig 2 expands on the portion of the tree in the neighborhood of the Poeciliidae. Divergence times for select nodes are provided in Table 1 based on autocorrelated rates (AUTO) and independent rates (IR). Analyses based on AUTO and IR provide similar estimates for the most recent common ancestor of Clupeocephala (AUTO = 163.8 Ma, IR = 163.1 Ma) and other deep nodes within this clade (Table 1). The most recent common ancestor of Cyprinodontiformes was estimated as 78.0 Ma (AUTO) to 90.7 Ma (IR). Estimates for the most recent common ancestor of Poeciliinae ranged from 53.4 Ma (AUTO) to 56.5 Ma (Table 1). Dates for other nodes that are relevant for understanding the biogeographic history of Poeciliinae (see below) are also provided in Table 1.

**Table 1:** Divergence times (Ma) taken from this and previous studies. Details on the calibrations used in each study can be found in the main text.

Clade	Study										
	This study (AUTO)	This study (IR)	Hrbek et al. (2007)	Alfaro et al. (2009)	Doadrio et al. (2009)	Santini et al. (2009)	Betancur-R et al. (2013)^A^	Alda et al. (2013)^B^	Ho et al. (2016)^C^	Palacios et al. (2016)^D^	Weaver et al. (2016)
Clupeocephala	163.8 (159.6–165.3)	163.1 (158.4–165.1)	NA	185.2	NA	173.8	250.6	NA	NA	NA	NA
Otocephala	154.2 (150.0–160.2)	153.2 (150.0–159.7)	NA	150.2	NA	150.6	230.2	NA	NA	NA	NA
Ostariophysi	144.2 (135.6–154.3)	139.9 (134.3–147.9)	NA	129.2	NA	128.3	198.6	NA	NA	NA	NA
Clupeomorpha	104.3 (84.5–142.2)	100.1 (83.6–120.4)	NA	NA	NA	91	87.1	NA	NA	NA	NA
Euteleostii (minus Lepidogalaxiidae)	161.4 (154.9–165.0	160.2 (151.0–164.6)	NA	173.7	NA	163.8	214.9	NA	NA	NA	NA
Tetraodontidae	42.4 (32.6–54.8)	44.5 (34.0–55.2)	NA	36.9	NA	38.3	35.6	NA	NA	NA	NA
Cyprinodontiformes	78.0 (39.6–96.5)	90.7 (84.5–97.1)	NA	NA	NA	NA	69.3	NA	NA	NA	NA
Cyprinodontidae to Poeciliidae	68.2 (36.1–78.7)	78.1 (70.9–84.0)	NA	NA	NA	NA	47.2	NA	NA	NA	NA
Cyprinodontidae (*Floridichthys* to *Jordanella*)	49.6 (29.0–62.3)	44.1 (30.5–58.7)	NA	NA	NA	NA	21.8	NA	NA	NA	NA
Anablepidae to Poeciliinae	62.4 (35.7–71.1)	66.9 (59.8–71.0)	NA	NA	NA	NA	NA	NA	NA	NA	NA
Poeciliinae	53.4 (28.9–63.2)	56.5 (50.1–62.6)	68	NA	NA	NA	NA	NA	NA	NA	NA
*Heterandria* to *Poecilia*	40.8 (22.3–50.7)	41.5 (37.3–46.0)	43.9	NA	NA	NA	17.4	NA	~12.5	NA	NA
*Poecilia*	30.5 (16.2–39.2)	26.4 (21.9–31.2)	NA	NA	NA	NA	NA	NA	~6	16.4/3.9	NA
*Mollienesia* to *Pamphorichthys + Limia*	25.5 (13.1–33.3)	20.1 (17.0–23.2)	21.9	NA	NA	NA	NA	~56/NA	NA	NA	NA
*Pamphorichthys* to *Limia*	24.1 (12.3–32.1)	19.1 (16.0–22.2)	19.9	NA	NA	NA	NA	NA	NA	NA	32.9
*Mollienesia*	19.1 (10.3–26.2)	14.3 (11.1–17.8)	NA	NA	NA	NA	NA	27.5/39.5	~3	5.8/1.3	NA
*Gambusia* to *Poeciliopsis*	37.2 (20.3–47.3)	37.5 (33.5–41.2)	43.9	NA	NA	NA	14.3	NA	NA	NA	NA
*Gambusia* to *Belonesox*	24.2 (13.3–32.0)	26.2 (21.4–30.5)	NA	NA	NA	NA	9.2	NA	NA	NA	NA
*Quintana* to *Girardinus*	25.9 (14.0–35.6)	25.5 (19.6–31.8)	NA	NA	~60	NA	NA	NA	NA	NA	NA

^A^ Divergence times based on updated data set provided by Guillermo Orti.

^B^ Divergence times before and after slashes correspond to analyses with 17–14 Ma and 25–20 Ma calibrations for *Limia melanonotata* to *L*. *vittata* assuming that this divergence reflects the vicariant separation of Hispaniola and Cuba (Wind Passage).

^C^ Approximate ages estimated from figures 2 and 3 of Ho et al. [23].

^D^ Divergence times before and after slashes correspond to analyses with a molecular clock using a cytochrome b rate of 1.0–2.0%, and a secondary fossil calibration applied to the origin of the genus *Poecilia* set to 6 Ma and a range of 2.5–9.5 Ma.

Abbreviations: AUTO = autocorrelated rates; IR = independent rates; NA = not applicable or not reported.

### Biogeographic analyses

Our tree topology replicates all of the 9 major clades within the Poeciliinae defined by Hrbek et al. [12], but adds details not present in the earlier analyses largely because of our broader representation of the order Cyprinodontiformes and additional species of Poeciliinae. One such detail is that our tree provides a robust definition of the sister taxa to Parenti’s Poeciliinae. Our phylogeny differs from Parenti’s [1, 11] by defining the Anablepidae as the sister taxa to Poeciliinae. Both of these taxa are exclusively New World clades. Parenti instead identified the subfamilies Fluviphylacinae (genus *Fluviphylax*–represented here by two species) and Aplocheilichtyinae (genus *Aplocheilichthys*, represented here by two species) as sister to the Poeciliinae. It was this sister relationship that motivated her to reclassify Rosen and Bailey’s [10] Poecillidae as one of three subfamilies of her newly defined Poeciliidae. In our tree, *Aplocheilichthys* is most closely allied with Valencidae. Both of these taxa are from the Old World and both are exclusively egg layers. We found that the Fluviphylacinae are sister to (Poeciliinae + Anablepidae). The Fluviphylacinae are composed of egg layers found in the Amazon basin. The closer affinity of Poeciliinae with Anablepidae and Fluviphylacinae, to the exclusion of Aplocheilichthyinae means that the radiation of these taxa likely took place on South America after it became isolated as an island continent. Our time tree analysis supports this conclusion, since the estimate for the node that represents the common ancestor of the Anablepidae, Poeciliinae and Fluviphylacinae dates to between 60 and 65 million years (Fig 2).

We applied additional scrutiny to our conclusion that Parenti’s [1] Poeciliinae and Anablepidae are sister taxa because this topology represents a departure from the conventional definition of the Poeciliidae [1, 11]. Statistical tests (Kishino-Hasegawa, Templeton, winning-sites) provide additional support for the hypothesis that Parenti’s [1, 11] Poeciliinae and Anablepidae are sister taxa and form a monophyletic clade to the exclusion of *Aplocheilichthys* and *Fluviphylax* (Table 2). All three topological resolutions of Parenti’s [1, 11] Poeciliidae required significantly more steps than Anablepidae + Poeciliinae, including tests that excluded the *X-SRC* gene that provided the basis for Meyer and Lydeard’s [89] original proposal of a sister-taxa relationship between the Poeciliinae and Anablepidae. Indeed, a tree patterned on Parenti’s hypothesis required 51 (with *X-SRC*) or 48 (without *X-SRC*) additional steps to constrain the monophyly of Parenti’s [1, 11] Poeciliidae relative to Meyer and Lydeard’s [89] hypothesis. We thus have strong confidence in the conclusion that the Anablepidae are sister to the Poeciliinae.

**Table 2:** Summary of statistical tests regarding the sister group to Poeciliinae.

Trees	Length	Length difference	Kishino-Hasegawa p value	Templeton p value	Winning-sites p value
1. Parenti’s [1] Poeciliinae plus Anablepidae	103475 (102135)	best tree			
2. Parenti’s [1] Poeciliidae:a. (F,(A,P)) b. ((F,A),P) c. (A,(F,P))	103537 (102189) 103526 (102183) 103537 (102191)	62 (54) 51 (48) 62 (56)	0.0001* (0.0005*) 0.0011* (0.0016*) 0.0001* (0.0003*)	0.0001* (0.0005*) 0.0012* (0.0017*) 0.0001* (0.0003*)	<0.0001* (0.0001*) 0.0003* (0.0004*) <0.0001* (0.0001*)

Statistical tests (Kishino-Hasegawa, Templeton, Winning-sites) were performed with PAUP* [90] and Pollux et al.’s [9] molecular data set to test prior hypotheses [1, 89] regarding the sister group to Poeciliinae. Values in parentheses are based on analyses without the *X-SRC* gene, which was included in Pollux et al.’s [9] data set and provided the basis for Meyer and Lydeard’s [89] original hypothesis that Anablepidae is the sister taxon to Parenti’s [1] Poeciliinae. Abbreviations: A, *Aplocheilichthys*; F, *Fluviphylax*, P, Poeciliinae. Asterisks indicate a significant difference at P = 0.01.

The results of biogeographic analyses are reported in Fig 2 (MAC parsimony) and Table 3 (DEC +J). We also performed an analysis with DEC, but this model was rejected by a likelihood ratio test (lnL DEC = -203.3, lnL DEC + J = -162.8, p = 2.3x10^-19^) and DEC results are not shown. Overall, MAC parsimony and DEC + J analyses yielded very similar results (Fig 2, Table 3), which strengthens our interpretation of the results reported below. Importantly, MAC parsimony and DEC + J both allow for dispersal to a new and unique area on a given branch. Biogeographic analyses strongly support a South American origin for the common ancestor of Poeciliinae and Anablepidae. Reconstructions of ancestral areas also confirm that Poeciliinae originated in the New World, most likely South America, and reveal details of the subsequent dispersal and radiation of this subfamily. Dispersal events are summarized in Table 4 and detailed in S3 Table. The alignment between our estimated divergence dates and those of Hrbek et al.[12] appear in Table 1.

**Table 3:** Probabilities for ancestral ranges based on DEC + J analyses.

Node Number (Fig 2)	Probabilities of Ancestral Areas
1	CA = 0.0778; NA = **0.9148**; CA+NA = 0.0074
2	CA = 0.0778; NA = **0.9147**; CA+NA = 0.0074
3	NA = 0.0419; WI = 0.1977; NA+WI = **0.7604**
4	CA = 0.0541; NA = **0.7162**; WI = 0.1367; CA+NA = 0.0030; NA + WI = 0.0896; CA+NA+WI = 0.0001
5	CA = **0.8026**; NA = 0.0395; WI = 0.1137; CA+NA = 0.0052; NA+WI = 0.0287; CA+WI = 0.0012; CA+NA+WI = 0.0090
6	CA = **0.7863**; WI = 0.2032; CA+WI = 0.0105
7	CA = **0.7895**; WI = 0.2041; CA+WI = 0.0065
8	CA = **0.7958**; WI = 0.1951; CA+WI = 0.0092
9	CA = **0.7909**; NA = 0.0157; WI = 0.1537; CA+NA = 0.0037; NA+WI = 0.0217; CA+WI = 0.0057; CA+NA+WI = 0.0086
10	CA = **0.7710**; NA = 0.0157; WI = 0.1734; CA+NA = 0.0034; NA+WI = 0.0208; CA+WI = 0.0072; CA+NA+WI = 0.0084
11	CA = 0.0223; NA = **0.9596**; CA+NA = 0.0181
12	CA = 0.0100; NA = **0.9773**; CA+NA = 0.0126
13	CA = **0.7591**; NA = 0.1165; WI = 0.0853; CA+NA = 0.0134; NA+WI = 0.0152; CA+WI = 0.0039; CA+NA+WI = 0.0066
14	CA = **0.9931**; NA = 0.0015; WI = 0.0011; CA+NA = 0.0025; CA+WI = 0.0014; CA+NA+WI = 0.0003
15	CA = **0.9627**; SA = 0.0121; CA+SA = 0.0253
16	CA = **0.9731**; NA = 0.0119; CA+NA = 0.0150
17	CA = **0.9666**; SA = 0.0120; CA+SA = 0.0213
18	CA = 0.4976; SA = 0.0029; WI = 0.4969; CA+SA = 0.0004; CA+WI = 0.0022
19	SA = **0.9842**; WI = 0.0125; SA+WI = 0.0033
20	CA = **0.9761**; NA = 0.0120; CA+NA = 0.0119
21	CA = 0.0123; SA = **0.9809**; WI = 0.0004; CA+SA = 0.0061; SA + WI = 0.0002
22	SA = **0.5002**; WI = 0.0001; SA+WI = 0.4997
23	CA = 0.0002; SA = **0.9795**; WI = 0.0002; CA+SA = 0.0020; SA+WI = 0.0180
24	CA = 0.0062; SA = **0.9773**; WI = 0.0063; CA+SA = 0.0055; SA+WI = 0.0045; CA+SA+WI = 0.0001
25	CA = 0.0880; SA = **0.8546**; CA+SA = 0.0572; CA+SA+WI = 0.0002

MAC parsimony reconstructions are denoted in bold. CA, Central America; NA, North America; SA, South America; WI, West Indies.

**Table 4:** Dispersal events in Poeciliinae based on MAC parsimony and DEC + J*.

To From	Central America	North America	South America	West Indies
Central America	-	4	2	3
North America	3	-	0	1
South America	2	0	-	1
West Indies	0	0	0	-

*Summary based on reconstructions for all nodes with unequivocal ancestral areas with MAC parsimony. See Fig 2 for single node (node 18) with equivocal reconstruction. In addition to dispersal events, two range expansions are supported by MAC parsimony—one from SA to SA + WI in *Poecilia vivipara* and one from CA to CA + NA in the ancestor of *Poeciliopsis occidentalis* + *Poeciliopsis sonoriensis*.

There were two dispersal events from South America to Central America. The first was the dispersal of the ancestor of *Xenodexia ctenolepis* (node 25 in Fig 2), dating to ≤53.4 Ma with AUTO and ≤ 56.4 Ma with IR. The second, more recent dispersal was the common ancestor of *Limia* + *Pamphorichthys* + *Mollienesia* (node 21) to the most recent common ancestor of subgenus *Mollienesia* (25.5–19.0 Ma, AUTO/20.1–14.3, IR).

There are two dispersal events associated with Node 18 that were ultimately derived from South America (node 24), but the path from South America through node 18 to its immediate descendant nodes cannot be resolved. Both events resulted in substantial radiations after dispersal. One dispersal event (40.8–37.2 Ma, AUTO/41.5–37.5 Ma, IR) leads to the most recent common ancestor of the Central American clade, which has radiated into the majority of extant species. The second dispersal event (40.8–25.9 Ma, AUTO/41.5–25.5 Ma, IR) gave rise to the common ancestor of the *Girardinus/Quintana* clade of the Greater Antilles. Our lack of resolution at node 18 means we cannot resolve whether the common ancestor of *Girardinus/Quintana* was derived from South or Central America. It also means we cannot resolve whether the common ancestor of the Central American radiation was derived from South America or the Greater Antilles. The timing of these colonization events is close to that reported by Hrbek et al. [12].

There was one dispersal from South America to the Caribbean which resulted in the radiation of the *Poecilia* subgenus *Limia* (Node 19, 22.8–16.2 Ma, AUTO/ 16.7–11.4 Ma, IR). One interesting feature of our tree pertains to the status of *Poecilia (Limia) heterandria*, a species whose subgeneric classification has varied over time. It has formerly been allied either with subgenus *Poecilia* or subgenus *Pamphorichthys*. Most recently, Poeser [91] classified it as its own genus (*Pseudolimia*). Our analysis shows that it is the basal lineage to the subgenus *Limia*, but is the only member of the subgenus found in South America.

There were nine later dispersal events out of Central America—two to South America, four to North America and three to the West Indies. The two dispersals from Central to South America resulted in *Neoheterandria* elegans (node 15, Ma ≤ 21.4 Ma, AUTO/≤ 21.7 Ma, IR) from Colombia and *Pseudopoecilia festae* (node 17, ≤ 24.4 Ma, AUTO/≤ 22.5 Ma, IR) from Colombia and Ecuador. These are the only representatives of the subfamily that have colonized the Pacific coast of South America. Ho et al. [23] report an additional dispersal event from Central to South American by species of *Poecilia*. This event is not present in our results because our data set lacked all *Poecilia* species derived from the ancestor that migrated from Central to South America.

The earliest colonization of North America is represented by *Heterandria formosa*, which is most closely related to *Priapichthys panamensis* (node 16) and arrived in North America ≤ 25.7 Ma (AUTO) or ≤ 22.8 Ma (IR). This inference, first made by Hrbek et al. [12], is of particular interest because Rosen and Baily [10] originally allied it with what is now classified as *Pseudoxiphophorus*, a genus represented by 12 species in Guatemala and Mexico [92].

The most recent dispersal events in our tree (nodes 1–14, <1.6 Ma– 11 Ma) all involve the genus *Gambusia*. These include three independent dispersals from Central America to North America and three from Central America to the Caribbean. The only dispersal events out of North America were three to Central America, again all by species in the genus *Gambusia*. There are also species in this genus that have ranges that span North America and the Caribbean.

## Discussion

Rosen and Bailey [10] opened their monograph with the observation that “The broad tolerance for brackish and saltwater environments of many of these chiefly freshwater species makes them of particular interest in zoogeographic studies and overwater dispersal…” In contrast, Rosen [13] emphasized the importance of vicariance and associated overland dispersal as key determinants of biogeographic distribution. Our results can be interpreted as support for both of these processes. Our results also confirm and extend those of Hrbek et al [12].

As inferred by Hrbek et al. [12], we found that the most recent common ancestor of Poeciliinae was found in South America, dating to the late Paleocene (56.5 Ma, IR) or early Eocene (53.4 Ma, AUTO). With the exception of *Xenodexia*, all early splitting lineages of the family (*Tomeurus*, *Phalloptychys*, *Phalloceros*, *Cnesterodon*, *Poecilia*) are exclusively found or are rooted in South America. We confirmed that the common ancestors of the taxa in the Greater Antilles (*Girardinus*, *Quintana*) and the large Central American radiations dispersed from South America approximately 45–40 Mya. These dates are consistent with dispersal over the Aves Land Bridge between South America and the nascent Greater Antilles [12]. Hrbek et al. [12] concluded that the common ancestor of the Central American radiation was derived from the Greater Antilles. Our analysis could not discriminate between the direct colonization of Central America from South America versus a two-step colonization process (first Greater Antilles, then Central America—Node 18 in Fig 2). Our and Hrbek et al’.s [12] analyses also agreed on the approximate timing of the dispersal of the common ancestor of the subgenus *Mollienesia* from South America to Central America (node 21), the subgenus *Limia* (save *L*. *heterandria*) from South America to the Greater Antilles, (Node 19), the dispersals out of Central America to North America by the genus *Gambusia* (Node 14) and to South America by *Pseudopoecilia festae* (Node 17) and *Neoheterandria* (Node 15). Our analysis revealed additional dispersals among North America, Central America and the Caribbean by members of the genus *Gambusia*, (Nodes 1–13). While arguments have been made for vicariance as a cause of the nodes between 14 and 25, at least some of those that lie between nodes 1 and 13 must represent dispersal through brackish water or marine barriers, made possible by the ability of many species of *Gambusia* to tolerate high salt concentrations. Palacios et al. [25] arrived at the same conclusion for the subgenus *Mollienesia* (Node 21) and the same could be true for *Limia* (Node 19). Species in both taxa can be found in brackish water and hence are tolerant of high salt concentrations [10, 93]. Either dispersal or vicariance represent plausible explanations for the origins of these nodes.

Our analyses confirmed Palacios et al.’s [25] finding of an explicit link between South America and the Caribbean for the *Poecilia* subgenus *Limia*. Prior investigators had inferred that *Limia* was derived from South America [12, 24] but their analyses did not include *Limia heterandria*. This inclusion reveals a monophyletic cluster of species that spans the distance between the presumed origin of the common ancestor of the genus and the Caribbean radiation. Palacios et al. [25] also show that a triad of Caribbean species (*Poecilia dominicensis*, *P*. *hispaniolae*, *P*. *elegans*—not included in our analyses) formerly classified with the subgenus *Mollienesia* are instead more closely allied to *Limia*. The fact that this clade of three species is basal to the entirety of *Limia* suggests that they might represent a second colonization of the Greater Antilles from South America. Alternatively, *L*. *heterandria* could represent a back dispersal from the Greater Antilles to South America.

Our work also reaffirms Hrbek et al’s[12] finding that *Heterandria formosa*, in the Southeastern United States, is most closely allied with *Priapichthys panamensis* in Central America This is a distribution that is difficult to reconcile with any form of vicariance. This association is coherent from the perspective of life histories. Both species have superfetation, or the ability to carry multiple broods of young in discretely different stages of development. The distribution of the trait suggests they inherited it from a common ancestor. *Heterandra formosa* also has matrotrophy, or the functional equivalent of a mammalian placenta. This distribution suggests that matrotrophy evolved in the *H*. *formosa* lineage after the evolution of superfetation. This same sequence of events happened three times in the genus *Poeciliopsis* [8] and once within the genus *Poecilia* [31, 94, 95].

### Timetree comparisons with other studies

Some of the studies of individual genera in the subfamily Poeciliinae yield time tree estimates for some of the same nodes contained in our tree. These estimates sometimes differ by a wide margin. Such variation invites a consideration of the means by which these estimates are arrived at and their efficacy. Here we consider some of this variation and some of the possible causes.

Our taxon sampling differs substantially from other time tree studies of bony fishes and necessarily includes its own unique set of calibrated nodes. For example, Betancur-R et al. [36] included a diverse assemblage of 1410 bony fishes plus four tetrapod and two chondrichthyan outgroups in their analyses whereas our analysis was limited to 293 taxa belonging to Clupeocephala. Betancur-R et al.'s [36] data set included 13 species in Cyprinodontiformes whereas our data set included 242 Cyprinodontiformes. Our estimated timetree dates also show both similarities and differences with Betancur-R et al. [36] and other studies (e.g., [12, 15, 20–25, 47, 96] (Table 1).

Divergence dates for several nodes that are deep in our tree, including Clupeocephala, Otocephala, Ostariophysi, and Euteleostii, are consistently younger than dates from Betancur-R et al. [36], but are comparable to dates reported by Alfaro et al. [46] and Santini et al. [47] (Table 1). We suspect that our younger dates result from constraining the root of the tree to have a maximum date of 165.2 Ma following Benton et al. [41]. As noted by Benton et al. [55], a maximum bound for Clupeocephala is highly arbitrary and a much older maximum is also possible. Betancur-R et al. [36] did not calibrate Clupeocephala, but calibrated several deeper nodes in their tree including Gnathostomata (519–426 Ma), Osteichthyes (438–418), Actinopterygii (423–398 Ma), and Actinopterii (415–375 Ma). This calibration strategy allowed Betancur-R et al.’s [36] estimated age for Clupeocephala to be significantly older than our estimated age.

Given our younger dates for deeper nodes, it is perhaps surprising that we recovered older dates within Cyprinodontiformes than Betancur-R et al. [36] (Table 1). Our estimates for the most recent common ancestor of Cyprinodontiformes are 78.0 and 90.7 Ma with autocorrelated and independent rates, respectively. Betancur-R et al.'s [36] date for this node is slightly younger at 69.3 Ma. Betancur-R et al.'s [36] dates for nodes within Cyprinodontiformes exhibit even more disagreement with our estimated dates. For example, Betancur-R et al.[36] estimated a divergence date of 21.8 Ma for Cyprinodontidae (*Floridichthys* to *Jordanella*) whereas our estimated dates for this node are ~2.5X larger at 44.1 to 49.6 Ma. Similarly, Betancur-R et al. [36] estimated that the Poeciliinae genera *Heterandria* and *Poecilia* last shared a common ancestor 17.4 Ma whereas our analyses recovered divergence time estimates of 40.8 Ma (AUTO) and 41.5 Ma (IR). Unlike Betancur-R et al. [36], who did not include any calibrations inside of Cyprinodontiformes, we included three calibrations (Fig 1, Node 5—Poeciliinae to Anablepidae; Node 15 –the Goodeidae; Node 16 –*Empetrichthys* to *Crenichthys*) in this order. The inclusion of these cyprinodontiform calibrations in our analysis may explain, at least in part, the different dates that were recovered in our analyses. Our data set also included a greater proportion of mitochondrial genes than Betancur-R et al [36], which adds resolution to the characterization of more recent nodes. Finally, as noted above, our taxon sampling for Cyprinodontiformes is much more dense than Betancur-R et al.'s [36] taxon sampling within this order.

Our dates for Poeciliinae and its subclades based on a fossil-calibrated tree are in good agreement with Hrbek et al.'s [12] dates for several overlapping nodes (Table 4). The similarities between the two studies is striking because there is little overlap in the DNA sequence data on which the trees were based and no overlap in way the tree was calibrated. Whereas we employed fossil calibrations to date our tree, Hrbek et al.[12] employed a secondary calibration from Reznick et al. [8], who in turn calibrated their tree for *Poeciliopsis* based on a vicariant event. What we have in common is extensive, subfamily-wide coverage.

Other studies have obtained both older and younger dates for divergences within Poeciliinae. Alda et al. [22] calibrated their tree based on alternate dates (25–20 Ma, 17–14 Ma) for the separation of Hispaniola versus Cuba (Wind Passage), which they tied to the cladogenetic separation of *Limia melanonotata* and *L*. *vittata*. Based on these alternate calibrations, Alda et al.[22] recovered dates that in some cases are more than twice as old as our estimated ages (Table 4). For example, our point estimates for *Mollienesia* to *Limia* are 20.1 and 25.5 Ma with independent and autocorrelated rates, respectively. By contrast, Alda et al. [22] obtained a date of 56 Ma for this split based on their 25–20 Ma calibration (date with 17–14 Ma calibration not reported). Doadrio et al [20] and Weaver et al. [24] used the 17–14 Ma date for the separation of Hispaniola and Cuba for their calibration of the cladogenic separation of *Limia vittata* from the remainder of the genus. Doadrio et al. [20] arrived at a point estimate of 62 Ma for the cladogenic separation of the common ancestor of *Girardinus/Quintana* from the Central American clade (node 18). We instead arrived at a point estimate of 40.8/41.5 Ma (autocorrelated/independent). Weaver et al. [23, 24] arrived at a point estimate of 32.9 Ma for the cladogenic separation of the *Poecilia* subgenus *Limia* from the *Poecilia* subgenus *Pamphorichthys*. We arrived at point estimates of 24.1/19.1 Ma (autocorrelated/independent) for this same event.

At the other extreme, Ho et al.[23] obtained much younger dates for various nodes within Poeciliinae and criticized previous analyses that employed biogeographic calibrations. More specifically, Ho et al.[23] suggest that inferences based on biogeographic calibrations always incur circular reasoning and should be avoided. However, this assessment is only logical if the estimated dates for a node used as a calibration point is subsequently employed to infer the age of the same vicariant event. Similarly, estimated ages for fossil-calibrated nodes have the potential to become circular if the estimated ages from a molecular dating analysis are championed as supporting the same divergence time as suggested by said fossils. Perhaps more importantly, Ho et al.'s [23] calibrated their timetree analysis with a single secondary calibration taken directly from Betancur-R et al. [36] for the separation of *Poecilia* and *Heterandria* (14.3 Ma). As noted above, Betancur-R et al.'s [36] analysis included far fewer Cyprinodontiformes than were included in our analysis (13 versus 242) and lacked calibrations within this clade.

Palacios et al. [25] took a different approach to developing a time tree, which was to use the “universal MtDNA mutation rate” to calibrate their tree for the *Poecilia* subgenus *Molliensia*. Their calibrations yielded consistently younger divergence dates than ours. For example, their estimated date of origin of the genus *Poecilia* was 16.4 Ma, as compared to our point estimates of 30.5/26.4 Ma (autocorrelated/independent).

Given widespread discordance among published studies for divergence times within Poeciliinae, we suggest that the true divergence dates remain to be determined. Our contribution is of value for what it reveals about the patterns of colonization and divergence, but what it can tell us about vicariance versus dispersal is limited by the uncertainty in the estimation of the timing of events. Future fossil discoveries loom as critical for gaining increased confidence in the timing of the Poeciliinae radiation. We also suggest that the time is ripe to perform simulation studies that will illuminate how the choice of calibration points and the nature of the data that serve as a basis for defining a phylogeny can jointly influence the values generated for a time tree.

## Supporting information

S1 Table Complete time trees which served as the basis for Fig 1 and Fig 2. (DOCX)

S2 Table Area assignments. (DOC)

S3 Table Details of colonization events summarized in Table 4. (DOCX)
