# Supplementary material for: The origin and biogeographic diversification of fishes in the family Poeciliidae
Source: PLoS One. 2017 Mar 9;12(3):e0172546. doi: 10.1371/journal.pone.0172546 (PMC5344339; doi:10.1371/journal.pone.0172546)
Supplement: S1 Table — (DOCX) [file pone.0172546.s001.docx]

S1 Table. Complete time trees which served as the basis for Fig 1 and Fig 2.

1. Timetree with 95% credibility intervals based on autocorrelated rates with hard-bounded constraints (1 unit = 100 million years)

(((((((((((((((((((((((((((((((((((((((((((Gambusia_hurtadoi: 0.044171, Gambusia_vittata: 0.044171) '0.019-0.076': 0.024685, (Gambusia_panuco: 0.043723, Gambusia_marshi: 0.043723) '0.011-0.080': 0.025133) '0.039-0.100': 0.011256, Gambusia_atrora: 0.080112) '0.047-0.113': 0.007000, (Gambusia_rhizophorae: 0.043678, Gambusia_punctata: 0.043678) '0.018-0.080': 0.043433) '0.052-0.122': 0.005224, Gambusia_zarskei: 0.092335) '0.055-0.129': 0.008425, ((((Gambusia_oligosticta: 0.003017, Gambusia_caymanensis: 0.003017) '0.000-0.010': 0.008234, Gambusia_puncticulata: 0.011252) '0.002-0.025': 0.022916, Gambusia_yucatana: 0.034168) '0.017-0.054': 0.031478, ((Gambusia_manni: 0.012202, Gambusia_hubbsi: 0.012202) '0.002-0.030': 0.042914, Gambusia_nicaraguensis: 0.055116) '0.028-0.085': 0.010530) '0.038-0.097': 0.035113) '0.061-0.140': 0.004199, ((Gambusia_melapleura: 0.036254, Gambusia_wrayi: 0.036254) '0.007-0.079': 0.027794, Gambusia_hispaniolae: 0.064048) '0.028-0.104': 0.040910) '0.064-0.146': 0.005250, (((Gambusia_affinis: 0.015911, Gambusia_sp_LLSTC4571: 0.015911) '0.007-0.027': 0.032995, Gambusia_holbrooki: 0.048906) '0.028-0.074': 0.048180, (Gambusia_geiseri: 0.054801, Gambusia_heterochir: 0.054801) '0.025-0.093': 0.042285) '0.059-0.137': 0.013122) '0.067-0.153': 0.031237, (Gambusia_eurystoma: 0.021000, Gambusia_sexradiata: 0.021000) '0.004-0.049': 0.120446) '0.085-0.193': 0.013894, Gambusia_luma: 0.155341) '0.091-0.210': 0.020799, (Heterophallus_milleri: 0.039888, Heterophallus_rachovii: 0.039888) '0.017-0.074': 0.136252) '0.100-0.235': 0.066206, Belonesox_belizanus: 0.242345) '0.133-0.320': 0.082949, (((((((((((Xiphophorus_couchianus: 0.012805, Xiphophorus_gordoni: 0.012805) '0.005-0.025': 0.004847, Xiphophorus_meyeri: 0.017653) '0.008-0.030': 0.033882, Xiphophorus_xiphidium: 0.051535) '0.024-0.084': 0.009001, Xiphophorus_variatus: 0.060536) '0.029-0.101': 0.003217, Xiphophorus_evelynae: 0.063753) '0.031-0.106': 0.019711, Xiphophorus_milleri: 0.083464) '0.040-0.136': 0.026132, Xiphophorus_maculatus: 0.109596) '0.054-0.165': 0.004322, Xiphophorus_andersi: 0.113918) '0.056-0.171': 0.008200, (((Xiphophorus_signum: 0.047146, Xiphophorus_mayae: 0.047146) '0.019-0.076': 0.010672, (Xiphophorus_alvarezi: 0.014400, Xiphophorus_hellerii: 0.014400) '0.005-0.029': 0.043418) '0.027-0.091': 0.058923, ((Xiphophorus_mixei: 0.041939, Xiphophorus_clemenciae: 0.041939) '0.014-0.080': 0.020848, Xiphophorus_monticolus: 0.062787) '0.028-0.137': 0.053953) '0.056-0.183': 0.005378) '0.060-0.184': 0.022663, ((((((Xiphophorus_nigrensis: 0.002776, Xiphophorus_multilineatus: 0.002776) '0.000-0.008': 0.000956, Xiphophorus_birchmanni: 0.003731) '0.000-0.009': 0.000873, Xiphophorus_continens: 0.004605) '0.001-0.011': 0.031379, ((Xiphophorus_cortezi: 0.019750, Xiphophorus_montezumae: 0.019750) '0.006-0.036': 0.010291, Xiphophorus_malinche: 0.030040) '0.009-0.062': 0.005943) '0.012-0.068': 0.014419, Xiphophorus_nezahualcoyotl: 0.050403) '0.019-0.086': 0.033244, Xiphophorus_pygmaeus: 0.083647) '0.031-0.131': 0.061134) '0.067-0.233': 0.163682, (Heterandria_bimaculata: 0.016470, Heterandria_jonesi: 0.016470) '0.005-0.031': 0.291992) '0.165-0.405': 0.016831) '0.175-0.427': 0.013311, ((Priapella_compressa: 0.044057, Priapella_chamulae: 0.044057) '0.019-0.075': 0.041364, (Priapella_olmecae: 0.068497, Priapella_intermedia: 0.068497) '0.036-0.100': 0.016924) '0.048-0.123': 0.253184) '0.183-0.440': 0.009874, ((Carlhubbsia_stuarti: 0.002004, Carlhubbsia_kidderi: 0.002004) '0.000-0.010': 0.324823, (Scolichthys_greenwayi: 0.013740, Scolichthys_iota: 0.013740) '0.003-0.035': 0.313087) '0.170-0.424': 0.021652) '0.189-0.455': 0.023090, ((((((((((Poeciliopsis_hnilickai: 0.012515, Poeciliopsis_catemaco: 0.012515) '0.006-0.021': 0.008785, Poeciliopsis_gracilis: 0.021300) '0.013-0.033': 0.007779, Poeciliopsis_pleurospilus: 0.029079) '0.016-0.050': 0.069033, (Poeciliopsis_presidionis: 0.062094, Poeciliopsis_turneri: 0.062094) '0.034-0.102': 0.036018) '0.058-0.142': 0.038463, (Poeciliopsis_scarlii: 0.041533, Poeciliopsis_turrubarensis: 0.041533) '0.018-0.069': 0.095042) '0.081-0.192': 0.035649, ((Poeciliopsis_latidens: 0.040757, Poeciliopsis_fasciata: 0.040757) '0.021-0.063': 0.036971, Poeciliopsis_baenschi: 0.077727) '0.044-0.118': 0.094497) '0.100-0.237': 0.017012, ((((((Poeciliopsis_occidentalis: 0.006485, Poeciliopsis_sonoriensis: 0.006485) '0.003-0.011': 0.021274, Poeciliopsis_lucida: 0.027759) '0.016-0.043': 0.007534, Poeciliopsis_prolifica: 0.035293) '0.021-0.052': 0.022707, Poeciliopsis_infans: 0.058000) '0.034-0.087': 0.090639, (Poeciliopsis_monacha: 0.095101, Poeciliopsis_viriosa: 0.095101) '0.049-0.149': 0.053538) '0.084-0.207': 0.008478, Poeciliopsis_balsas: 0.157118) '0.088-0.216': 0.032118) '0.109-0.259': 0.127170, ((Poeciliopsis_retropinna: 0.176690, Poeciliopsis_elongata: 0.176690) '0.094-0.246': 0.031592, Poeciliopsis_paucimaculata: 0.208282) '0.115-0.286': 0.108123) '0.174-0.405': 0.019392, ((Neoheterandria_cana: 0.025324, Neoheterandria_tridentiger: 0.025324) '0.009-0.051': 0.188594, Neoheterandria_elegans: 0.213918) '0.112-0.292': 0.121880) '0.182-0.430': 0.023503, ((((((((((Brachyrhaphis_terrabensis: 0.086368, Brachyrhaphis_roseni: 0.086368) '0.037-0.152': 0.032521, Brachyrhaphis_rhabdophora: 0.118889) '0.060-0.176': 0.068654, Brachyrhaphis_holdridgei: 0.187543) '0.097-0.261': 0.034169, Brachyrhaphis_hartwegi: 0.221712) '0.117-0.302': 0.026173, (((Phallichthys_amates: 0.029453, Phallichthys_pittieri: 0.029453) '0.006-0.074': 0.189165, Phallichthys_quadripunctatus: 0.218618) '0.115-0.294': 0.011807, Phallichthys_tico: 0.230425) '0.122-0.311': 0.017460) '0.133-0.327': 0.007686, ((Brachyrhaphis_parismina: 0.009297, Brachyrhaphis_cascajalensis: 0.009297) '0.001-0.025': 0.210686, (Priapichthys_puetzi: 0.118899, Priapichthys_annectens: 0.118899) '0.055-0.206': 0.101084) '0.117-0.329': 0.035588) '0.136-0.338': 0.025276, (Alfaro_hubberi: 0.144015, Alfaro_cultratus: 0.144015) '0.033-0.289': 0.136833) '0.151-0.366': 0.009943, Xenophallus_umbratilis: 0.290790) '0.156-0.382': 0.041923, (Priapichthys_panamensis: 0.256889, Heterandria_formosa: 0.256889) '0.141-0.353': 0.075825) '0.180-0.423': 0.013115, (Priapichthys_darienensis: 0.243699, Pseudopoecilia_festae: 0.243699) '0.136-0.337': 0.102129) '0.186-0.440': 0.013472) '0.197-0.457': 0.012268) '0.203-0.473': 0.025341, (((((((Girardinus_sp_GMIC19: 0.009978, Girardinus_rivasi: 0.009978) '0.003-0.021': 0.023493, Girardinus_microdactylus: 0.033471) '0.015-0.052': 0.037119, Girardinus_metallicus: 0.070590) '0.035-0.121': 0.038029, Girardinus_creolus: 0.108619) '0.060-0.160': 0.010919, (Glaridichthys_falcatus: 0.058303, Glaridichthys_uninotatus: 0.058303) '0.028-0.093': 0.061235) '0.065-0.178': 0.056746, ((Dactylophallus_sp_DDEN10: 0.021685, Dactylophallus_ramsdeni: 0.021685) '0.009-0.035': 0.004034, Dactylophallus_denticulatus: 0.025719) '0.011-0.041': 0.150565) '0.098-0.252': 0.082277, Quintana_atrizona: 0.258561) '0.140-0.356': 0.138349) '0.216-0.497': 0.010895, (((((((((((((((Limia_rivasi: 0.000414, Limia_tridens: 0.000414) '0.000-0.001': 0.000380, Limia_melanonotata: 0.000795) '0.000-0.002': 0.000251, Limia_perugiae: 0.001045) '0.000-0.002': 0.001111, Limia_sulfurophila: 0.002157) '0.000-0.006': 0.028009, ((Limia_garnieri: 0.002547, Limia_grossidens: 0.002547) '0.000-0.007': 0.001215, Limia_nigrofasciata: 0.003762) '0.001-0.008': 0.026404) '0.011-0.046': 0.044275, (Limia_dominicensis: 0.037857, Limia_pauciradiata: 0.037857) '0.014-0.060': 0.036583) '0.026-0.104': 0.041527, (Limia_vittata: 0.040390, Limia_caymanensis: 0.040390) '0.014-0.073': 0.075578) '0.047-0.157': 0.032304, (Limia_versicolor: 0.079331, Limia_zonata: 0.079331) '0.027-0.124': 0.068940) '0.065-0.198': 0.013449, Limia_melanogaster: 0.161720) '0.072-0.215': 0.066004, Limia_heterandria: 0.227724) '0.113-0.315': 0.013298, (((Pamphorichthys_hollandi: 0.062976, Pamphorichthys_araguaiensis: 0.062976) '0.030-0.102': 0.029667, Pamphorichthys_hasemani: 0.092643) '0.048-0.133': 0.019007, (Pamphorichthys_minor: 0.057289, Pamphorichthys_scalpridens_Sant: 0.057289) '0.025-0.114': 0.054361) '0.059-0.159': 0.129372) '0.123-0.321': 0.013636, (((((((((Poecilia_mexicana_lim: 0.028335, Poecilia_gracilis: 0.028335) '0.014-0.057': 0.005287, Poecilia_sulphuraria: 0.033623) '0.019-0.062': 0.015919, ((Poecilia_sphenops: 0.017343, Poecilia_catemaconis: 0.017343) '0.006-0.033': 0.026459, Poecilia_mexicana_mex: 0.043802) '0.021-0.089': 0.005740) '0.028-0.098': 0.003049, (Poecilia_gilli: 0.048390, Poecilia_salvatoris: 0.048390) '0.026-0.097': 0.004201) '0.030-0.101': 0.009856, Poecilia_orri: 0.062447) '0.038-0.115': 0.031374, Poecilia_butleri_MR04: 0.093821) '0.056-0.147': 0.066326, Poecilia_chica: 0.160147) '0.087-0.225': 0.010348, (((Poecilia_petenensis_MP523: 0.002920, Poecilia_petenensis_Campeche: 0.002920) '0.000-0.008': 0.087895, Poecilia_latipunctata: 0.090814) '0.047-0.140': 0.022995, (Poecilia_velifera_MP737: 0.063412, Poecilia_latipinna: 0.063412) '0.028-0.106': 0.050398) '0.065-0.172': 0.056685) '0.092-0.238': 0.019608, Poecilia_caucana: 0.190102) '0.103-0.262': 0.064555) '0.131-0.333': 0.021897, (Poecilia_vivipara: 0.044162, Poecilia_vivipara_Trinidad: 0.044162) '0.016-0.092': 0.232392) '0.146-0.357': 0.028598, (((((Micropoecilia_sarrafae: 0.022433, Micropoecilia_minima: 0.022433) '0.010-0.038': 0.067246, Micropoecilia_bifurca: 0.089679) '0.046-0.155': 0.064003, (Micropoecilia_parae_Def: 0.007847, Micropoecilia_parae: 0.007847) '0.002-0.020': 0.145836) '0.083-0.209': 0.045882, (Micropoecilia_picta_Trin: 0.028347, Micropoecilia_picta: 0.028347) '0.011-0.058': 0.171217) '0.105-0.268': 0.038547, ((Micropoecilia_reticulata: 0.005244, Micropoecilia_obscura: 0.005244) '0.000-0.012': 0.047251, (Micropoecilia_wingei_Fel: 0.005392, Micropoecilia_wingei: 0.005392) '0.001-0.010': 0.047104) '0.025-0.103': 0.185617) '0.124-0.314': 0.067040) '0.162-0.392': 0.052580, ((Cnesterodon_septentrionalis: 0.088904, Cnesterodon_decemmaculatus: 0.088904) '0.050-0.130': 0.028012, Cnesterodon_hypselurus: 0.116916) '0.067-0.177': 0.240817) '0.194-0.450': 0.050073) '0.223-0.507': 0.019347, Phalloceros_caudimaculatus: 0.427152) '0.234-0.528': 0.006139, Phalloptychus_januarius: 0.433291) '0.237-0.535': 0.049190, Tomeurus_gracilis: 0.482481) '0.266-0.586': 0.050971, Xenodexia_ctenolepis: 0.533452) '0.289-0.632': 0.090461, (((Jenynsia_multidentata: 0.013434, Jenynsia_lineata: 0.013434) '0.002-0.042': 0.561708, (Anableps_dovii: 0.006481, Anableps_anableps: 0.006481) '0.001-0.024': 0.568661) '0.326-0.670': 0.023184, Oxyzygonectes_dovii: 0.598326) '0.331-0.693': 0.025587) '0.357-0.711': 0.019689, (Fluviphylax_simplex: 0.188584, Fluviphylax_pygmaeus: 0.188584) '0.064-0.375': 0.455019) '0.359-0.738': 0.016868, (((Aplocheilichthys_normani: 0.380067, Aplocheilichthys_spilauchen: 0.380067) '0.061-0.595': 0.227577, Valencia_hispanica: 0.607644) '0.321-0.718': 0.027359, Orestias: 0.635003) '0.327-0.743': 0.025467) '0.359-0.760': 0.021136, (((((((((((((((((Allotoca_diazi: 0.004741, Allotoca_meeki: 0.004741) '0.001-0.010': 0.003735, Allotoca_catarinae: 0.008476) '0.004-0.016': 0.016873, Allotoca_zacapuensis: 0.025349) '0.013-0.043': 0.023542, (Allotoca_dugesii: 0.041439, Allotoca_goslinei: 0.041439) '0.027-0.059': 0.007452) '0.034-0.068': 0.014400, Allotoca_sp_MNCN3676: 0.063291) '0.043-0.088': 0.012182, Allotoca_maculata: 0.075473) '0.055-0.103': 0.028987, Allotoca_regalis: 0.104460) '0.082-0.135': 0.011356, Hubbsina_turneri: 0.115816) '0.092-0.148': 0.006520, ((((Skiffia_francesae: 0.007416, Skiffia_multipunctata: 0.007416) '0.003-0.014': 0.030219, Skiffia_lermae: 0.037635) '0.024-0.059': 0.052917, Skiffia_bilineatus: 0.090552) '0.070-0.118': 0.014282, ((Girardinichthys_multiradiatus: 0.069390, Girardinichthys_viviparus: 0.069390) '0.045-0.098': 0.029957, Neotoca_bilineata: 0.099347) '0.079-0.128': 0.005486) '0.085-0.134': 0.017502) '0.098-0.155': 0.013972, Ataeniobius_toweri: 0.136308) '0.112-0.170': 0.007575, (((((((Chapalichthys_pardalis: 0.007340, Chapalichthys_encaustus: 0.007340) '0.001-0.015': 0.036166, Alloophorus_robustus: 0.043506) '0.031-0.063': 0.004721, Xenotoca_variatus: 0.048227) '0.035-0.068': 0.007445, Ameca_splendens: 0.055671) '0.041-0.078': 0.038823, ((Zoogoneticus_quitzeoensis: 0.062124, Zoogoneticus_tequila: 0.062124) '0.039-0.085': 0.027411, Xenoophorus_captiva: 0.089535) '0.071-0.116': 0.004960) '0.076-0.122': 0.006094, (Xenotoca_melanosoma: 0.051250, Xenotoca_eiseni: 0.051250) '0.025-0.083': 0.049339) '0.081-0.129': 0.024458, (Goodea_gracilis: 0.003623, Goodea_atripinnis: 0.003623) '0.000-0.011': 0.121423) '0.102-0.155': 0.018837) '0.119-0.177': 0.011598, ((((Ilyodon_whitei: 0.004566, Ilyodon_xantusi: 0.004566) '0.001-0.013': 0.014016, (Ilyodon_furcidens: 0.010720, Ilyodon_amecae: 0.010720) '0.003-0.021': 0.007862) '0.010-0.032': 0.066140, Xenotaenia_resolanae: 0.084722) '0.060-0.116': 0.033641, (((Allodontichthys_hubbsi: 0.023218, Allodontichthys_tamazulae: 0.023218) '0.012-0.047': 0.004011, Allodontichthys_polylepis: 0.027229) '0.016-0.049': 0.003764, Allodontichthys_zonistius: 0.030993) '0.018-0.053': 0.087370) '0.091-0.151': 0.037118) '0.130-0.186': 0.017757, (Characodon_lateralis: 0.019066, Characodon_audax: 0.019066) '0.007-0.039': 0.154172) '0.144-0.210': 0.049505, ((Crenichthys_baileyi: 0.063317, Crenichthys_nevadae: 0.063317) '0.033-0.096': 0.054079, Empetrichthys_latos: 0.117396) '0.082-0.179': 0.105347) '0.200-0.230': 0.171311, (Profundulus_punctatus: 0.089920, Profundulus_guatemalensis: 0.089920) '0.028-0.199': 0.304135) '0.280-0.497': 0.051623, Profundulus_labialis: 0.445678) '0.296-0.552': 0.227401, (((Lucania_goodei: 0.015973, Lucania_parvae: 0.015973) '0.004-0.040': 0.324787, (Fundulus_lineolatus: 0.189434, Fundulus_cingulatus: 0.189434) '0.051-0.310': 0.151326) '0.171-0.470': 0.274942, ((Jordanella_floridae: 0.307752, Cyprinodon_variegatus: 0.307752) '0.084-0.491': 0.188175, Floridichthys_carpio: 0.495928) '0.290-0.623': 0.119774) '0.316-0.730': 0.057376) '0.359-0.778': 0.008528) '0.361-0.787': 0.025886, (Cubanichthys_pengelleyi: 0.264560, Cubanichthys_cubensis: 0.264560) '0.077-0.480': 0.442932) '0.366-0.822': 0.072369, (((((Aphyosemion_bitaeniatum: 0.258039, Fundulopanchax: 0.258039) '0.122-0.379': 0.074805, Adamas_formosus: 0.332844) '0.159-0.470': 0.194576, (Aphyoplatys_duboisi: 0.265052, Epiplatys_annulatus: 0.265052) '0.076-0.438': 0.262368) '0.288-0.669': 0.099897, Aplocheilus_lineatus: 0.627317) '0.311-0.764': 0.070548, Rivulus_hartii: 0.697865) '0.374-0.836': 0.081996) '0.378-0.912': 0.040887, (Oryzias_latipes: 0.700186, Atheriniformes: 0.700186) '0.140-0.877': 0.120562) '0.396-0.965': 0.034283, (Oreochromis: 0.631887, Cichlasomatinae: 0.631887) '0.397-0.837': 0.223144) '0.398-1.010': 0.018507, Mugil: 0.873538) '0.398-1.033': 0.051851, (Pleuronectiformes: 0.866250, Monopterus_albus: 0.866250) '0.398-1.043': 0.059139) '0.399-1.105': 0.015466, ((((Tetraodon_nigroviridis: 0.423703, Takifugu_rubripes: 0.423703) '0.326-0.548': 0.426334, Lophius: 0.850036) '0.354-1.030': 0.000000, (Lutjanus: 0.711036, Morone: 0.711036) '0.354-0.840': 0.139001) '0.354-1.030': 0.000000, ((Lycodes: 0.482585, Gasterosteus_aculeatus: 0.482585) '0.156-0.732': 0.283695, Scorpaeniformes: 0.766280) '0.348-0.943': 0.083757) '0.354-1.030': 0.090819) '0.399-1.125': 0.049136, (Ophidiiformes: 0.710877, Porichthys: 0.710877) '0.143-1.019': 0.279115) '0.400-1.189': 0.107221, Beryciformes: 1.097212) '0.508-1.292': 0.095710, Lampriformes: 1.192922) '0.510-1.383': 0.058480, (Polymixia: 1.086223, Zeus_faber: 1.086223) '0.076-1.363': 0.165179) '0.768-1.405': 0.033351, Myctophiformes: 1.284753) '0.768-1.440': 0.128066, Synodus: 1.412820) '1.277-1.503': 0.034689, ((Gadus_morhua: 0.946500, Macrouridae: 0.946500) '0.661-1.169': 0.442611, Aphredoderus_sayanus: 1.389111) '1.247-1.483': 0.058397) '1.328-1.525': 0.141438, (Osmeriformes: 1.381200, Stomiiformes: 1.381200) '0.438-1.570': 0.207746) '1.522-1.639': 0.024942, ((Oncorhynchus_mykiss: 1.075018, Esox_lucius: 1.075018) '0.731-1.252': 0.409922, Argentina: 1.484940) '1.306-1.619': 0.128949) '1.549-1.650': 0.023566, (((((Semotilus_atromaculatus: 0.289748, Notemigonus_crysoleucas: 0.289748) '0.090-0.581': 0.477776, Danio_rerio: 0.767524) '0.380-1.241': 0.606346, ((Characiformes: 1.113368, Ictalurus_punctatus: 1.113368) '0.948-1.251': 0.094860, Gymnotiformes: 1.208228) '1.054-1.424': 0.165642) '1.269-1.507': 0.068407, Chanos_chanos: 1.442277) '1.356-1.543': 0.099443, ((Pellona: 0.835812, Dorosoma_cepedianum: 0.835812) '0.253-1.136': 0.207015, Chirocentrus_dorab: 1.042827) '0.845-1.422': 0.498893) '1.500-1.602': 0.095734) '1.596-1.653': 0.002665;

2. Timetree with 95% credibility intervals based on independent rates rates with hard-bounded constraints (1 unit = 100 million years)

(((((((((((((((((((((((((((((((((((((((((((Gambusia_hurtadoi: 0.043900, Gambusia_vittata: 0.043900) '0.009-0.077': 0.029789, (Gambusia_panuco: 0.040842, Gambusia_marshi: 0.040842) '0.008-0.074': 0.032847) '0.043-0.107': 0.016164, Gambusia_atrora: 0.089853) '0.059-0.120': 0.013806, (Gambusia_rhizophorae: 0.050796, Gambusia_punctata: 0.050796) '0.027-0.080': 0.052863) '0.069-0.135': 0.010269, Gambusia_zarskei: 0.113929) '0.078-0.145': 0.012860, ((((Gambusia_oligosticta: 0.006281, Gambusia_caymanensis: 0.006281) '0.001-0.019': 0.014730, Gambusia_puncticulata: 0.021010) '0.006-0.045': 0.025883, Gambusia_yucatana: 0.046893) '0.025-0.074': 0.030843, ((Gambusia_manni: 0.016290, Gambusia_hubbsi: 0.016290) '0.002-0.042': 0.043765, Gambusia_nicaraguensis: 0.060055) '0.029-0.097': 0.017681) '0.049-0.115': 0.049052) '0.098-0.155': 0.006427, ((Gambusia_melapleura: 0.039715, Gambusia_wrayi: 0.039715) '0.013-0.072': 0.037645, Gambusia_hispaniolae: 0.077360) '0.035-0.130': 0.055856) '0.106-0.161': 0.008051, (((Gambusia_affinis: 0.025526, Gambusia_sp_LLSTC4571: 0.025526) '0.011-0.048': 0.033736, Gambusia_holbrooki: 0.059262) '0.036-0.091': 0.057149, (Gambusia_geiseri: 0.064367, Gambusia_heterochir: 0.064367) '0.029-0.116': 0.052044) '0.074-0.155': 0.024855) '0.113-0.169': 0.024165, (Gambusia_eurystoma: 0.026584, Gambusia_sexradiata: 0.026584) '0.002-0.066': 0.138847) '0.132-0.198': 0.016123, Gambusia_luma: 0.181554) '0.147-0.214': 0.020869, (Heterophallus_milleri: 0.050508, Heterophallus_rachovii: 0.050508) '0.024-0.087': 0.151915) '0.167-0.236': 0.059071, Belonesox_belizanus: 0.261494) '0.214-0.305': 0.057584, (((((((((((Xiphophorus_couchianus: 0.007334, Xiphophorus_gordoni: 0.007334) '0.003-0.013': 0.002565, Xiphophorus_meyeri: 0.009899) '0.005-0.016': 0.014281, Xiphophorus_xiphidium: 0.024180) '0.016-0.034': 0.006187, Xiphophorus_variatus: 0.030367) '0.021-0.041': 0.001787, Xiphophorus_evelynae: 0.032154) '0.023-0.043': 0.009414, Xiphophorus_milleri: 0.041568) '0.029-0.056': 0.015771, Xiphophorus_maculatus: 0.057339) '0.045-0.072': 0.003910, Xiphophorus_andersi: 0.061249) '0.048-0.077': 0.007385, (((Xiphophorus_signum: 0.020601, Xiphophorus_mayae: 0.020601) '0.005-0.037': 0.012399, (Xiphophorus_alvarezi: 0.009318, Xiphophorus_hellerii: 0.009318) '0.004-0.017': 0.023681) '0.022-0.047': 0.030960, ((Xiphophorus_mixei: 0.024090, Xiphophorus_clemenciae: 0.024090) '0.006-0.047': 0.012389, Xiphophorus_monticolus: 0.036480) '0.018-0.057': 0.027479) '0.051-0.080': 0.004675) '0.056-0.085': 0.026877, ((((((Xiphophorus_nigrensis: 0.003712, Xiphophorus_multilineatus: 0.003712) '0.001-0.009': 0.000866, Xiphophorus_birchmanni: 0.004578) '0.001-0.010': 0.001170, Xiphophorus_continens: 0.005748) '0.002-0.011': 0.015170, ((Xiphophorus_cortezi: 0.013356, Xiphophorus_montezumae: 0.013356) '0.007-0.022': 0.004036, Xiphophorus_malinche: 0.017392) '0.009-0.026': 0.003526) '0.013-0.030': 0.007322, Xiphophorus_nezahualcoyotl: 0.028240) '0.018-0.041': 0.025014, Xiphophorus_pygmaeus: 0.053254) '0.036-0.074': 0.042257) '0.073-0.127': 0.199138, (Heterandria_bimaculata: 0.019669, Heterandria_jonesi: 0.019669) '0.010-0.035': 0.274980) '0.235-0.340': 0.024429) '0.273-0.358': 0.016475, ((Priapella_compressa: 0.036854, Priapella_chamulae: 0.036854) '0.018-0.061': 0.032247, (Priapella_olmecae: 0.055582, Priapella_intermedia: 0.055582) '0.035-0.082': 0.013519) '0.048-0.099': 0.266452) '0.291-0.373': 0.010998, ((Carlhubbsia_stuarti: 0.002401, Carlhubbsia_kidderi: 0.002401) '0.000-0.008': 0.316573, (Scolichthys_greenwayi: 0.013720, Scolichthys_iota: 0.013720) '0.005-0.029': 0.305254) '0.263-0.364': 0.027577) '0.304-0.384': 0.028217, ((((((((((Poeciliopsis_hnilickai: 0.012810, Poeciliopsis_catemaco: 0.012810) '0.006-0.023': 0.011371, Poeciliopsis_gracilis: 0.024181) '0.014-0.038': 0.014784, Poeciliopsis_pleurospilus: 0.038965) '0.021-0.066': 0.073523, (Poeciliopsis_presidionis: 0.059873, Poeciliopsis_turneri: 0.059873) '0.036-0.094': 0.052615) '0.079-0.149': 0.040161, (Poeciliopsis_scarlii: 0.056636, Poeciliopsis_turrubarensis: 0.056636) '0.032-0.090': 0.096013) '0.118-0.190': 0.047085, ((Poeciliopsis_latidens: 0.049039, Poeciliopsis_fasciata: 0.049039) '0.026-0.080': 0.035305, Poeciliopsis_baenschi: 0.084344) '0.051-0.128': 0.115389) '0.166-0.235': 0.019981, ((((((Poeciliopsis_occidentalis: 0.013107, Poeciliopsis_sonoriensis: 0.013107) '0.005-0.028': 0.026371, Poeciliopsis_lucida: 0.039477) '0.024-0.059': 0.011125, Poeciliopsis_prolifica: 0.050602) '0.033-0.072': 0.029945, Poeciliopsis_infans: 0.080547) '0.054-0.116': 0.087962, (Poeciliopsis_monacha: 0.098940, Poeciliopsis_viriosa: 0.098940) '0.053-0.153': 0.069569) '0.132-0.206': 0.012921, Poeciliopsis_balsas: 0.181430) '0.146-0.217': 0.038285) '0.188-0.259': 0.094780, ((Poeciliopsis_retropinna: 0.155163, Poeciliopsis_elongata: 0.155163) '0.097-0.212': 0.034640, Poeciliopsis_paucimaculata: 0.189802) '0.130-0.247': 0.124693) '0.275-0.356': 0.020734, ((Neoheterandria_cana: 0.019937, Neoheterandria_tridentiger: 0.019937) '0.009-0.039': 0.196531, Neoheterandria_elegans: 0.216469) '0.156-0.289': 0.118761) '0.297-0.375': 0.027376, ((((((((((Brachyrhaphis_terrabensis: 0.051508, Brachyrhaphis_roseni: 0.051508) '0.019-0.100': 0.042195, Brachyrhaphis_rhabdophora: 0.093703) '0.058-0.136': 0.042608, Brachyrhaphis_holdridgei: 0.136311) '0.075-0.194': 0.046562, Brachyrhaphis_hartwegi: 0.182873) '0.147-0.217': 0.022749, (((Phallichthys_amates: 0.025661, Phallichthys_pittieri: 0.025661) '0.008-0.055': 0.150791, Phallichthys_quadripunctatus: 0.176452) '0.138-0.210': 0.013729, Phallichthys_tico: 0.190181) '0.155-0.223': 0.015441) '0.173-0.240': 0.009582, ((Brachyrhaphis_parismina: 0.007045, Brachyrhaphis_cascajalensis: 0.007045) '0.001-0.019': 0.159620, (Priapichthys_puetzi: 0.114230, Priapichthys_annectens: 0.114230) '0.055-0.177': 0.052436) '0.106-0.215': 0.048538) '0.182-0.251': 0.021612, (Alfaro_hubberi: 0.111409, Alfaro_cultratus: 0.111409) '0.024-0.221': 0.125407) '0.198-0.282': 0.017455, Xenophallus_umbratilis: 0.254270) '0.210-0.301': 0.053196, (Priapichthys_panamensis: 0.228146, Heterandria_formosa: 0.228146) '0.159-0.300': 0.079320) '0.260-0.354': 0.026890, (Priapichthys_darienensis: 0.224572, Pseudopoecilia_festae: 0.224572) '0.146-0.310': 0.109784) '0.286-0.378': 0.028250) '0.324-0.401': 0.012163) '0.335-0.412': 0.025906, (((((((Girardinus_sp_GMIC19: 0.012287, Girardinus_rivasi: 0.012287) '0.004-0.024': 0.016612, Girardinus_microdactylus: 0.028899) '0.015-0.047': 0.036414, Girardinus_metallicus: 0.065313) '0.043-0.096': 0.038946, Girardinus_creolus: 0.104259) '0.072-0.140': 0.017217, (Glaridichthys_falcatus: 0.052625, Glaridichthys_uninotatus: 0.052625) '0.030-0.081': 0.068850) '0.089-0.161': 0.053470, ((Dactylophallus_sp_DDEN10: 0.020547, Dactylophallus_ramsdeni: 0.020547) '0.009-0.035': 0.004902, Dactylophallus_denticulatus: 0.025449) '0.013-0.042': 0.149497) '0.133-0.221': 0.080023, Quintana_atrizona: 0.254968) '0.196-0.318': 0.145707) '0.359-0.444': 0.014564, (((((((((((((((Limia_rivasi: 0.000501, Limia_tridens: 0.000501) '0.000-0.002': 0.000386, Limia_melanonotata: 0.000887) '0.000-0.002': 0.000512, Limia_perugiae: 0.001399) '0.000-0.003': 0.005171, Limia_sulfurophila: 0.006571) '0.001-0.015': 0.018597, ((Limia_garnieri: 0.001690, Limia_grossidens: 0.001690) '0.000-0.005': 0.001195, Limia_nigrofasciata: 0.002885) '0.001-0.007': 0.022283) '0.014-0.039': 0.023315, (Limia_dominicensis: 0.028250, Limia_pauciradiata: 0.028250) '0.015-0.044': 0.020232) '0.034-0.065': 0.022563, (Limia_vittata: 0.024384, Limia_caymanensis: 0.024384) '0.011-0.043': 0.046662) '0.053-0.096': 0.028038, (Limia_versicolor: 0.054801, Limia_zonata: 0.054801) '0.033-0.082': 0.044283) '0.075-0.128': 0.014381, Limia_melanogaster: 0.113464) '0.088-0.144': 0.053424, Limia_heterandria: 0.166889) '0.132-0.203': 0.024137, (((Pamphorichthys_hollandi: 0.040923, Pamphorichthys_araguaiensis: 0.040923) '0.023-0.065': 0.037672, Pamphorichthys_hasemani: 0.078595) '0.053-0.113': 0.021617, (Pamphorichthys_minor: 0.062311, Pamphorichthys_scalpridens_Sant: 0.062311) '0.037-0.100': 0.037900) '0.072-0.135': 0.090814) '0.160-0.222': 0.009994, (((((((((Poecilia_mexicana_lim: 0.018251, Poecilia_gracilis: 0.018251) '0.009-0.030': 0.003408, Poecilia_sulphuraria: 0.021659) '0.012-0.033': 0.012668, ((Poecilia_sphenops: 0.012131, Poecilia_catemaconis: 0.012131) '0.005-0.021': 0.016580, Poecilia_mexicana_mex: 0.028711) '0.019-0.040': 0.005616) '0.024-0.046': 0.005366, (Poecilia_gilli: 0.035094, Poecilia_salvatoris: 0.035094) '0.022-0.048': 0.004600) '0.029-0.052': 0.007923, Poecilia_orri: 0.047617) '0.035-0.061': 0.012847, Poecilia_butleri_MR04: 0.060464) '0.045-0.079': 0.046066, Poecilia_chica: 0.106530) '0.079-0.140': 0.011578, (((Poecilia_petenensis_MP523: 0.002055, Poecilia_petenensis_Campeche: 0.002055) '0.000-0.005': 0.047997, Poecilia_latipunctata: 0.050052) '0.029-0.075': 0.020168, (Poecilia_velifera_MP737: 0.036258, Poecilia_latipinna: 0.036258) '0.019-0.058': 0.033963) '0.049-0.100': 0.047887) '0.089-0.152': 0.024654, Poecilia_caucana: 0.142762) '0.111-0.178': 0.058258) '0.170-0.233': 0.027469, (Poecilia_vivipara: 0.031628, Poecilia_vivipara_Trinidad: 0.031628) '0.015-0.056': 0.196861) '0.192-0.274': 0.035717, (((((Micropoecilia_sarrafae: 0.028123, Micropoecilia_minima: 0.028123) '0.014-0.049': 0.059962, Micropoecilia_bifurca: 0.088085) '0.054-0.128': 0.056894, (Micropoecilia_parae_Def: 0.010378, Micropoecilia_parae: 0.010378) '0.004-0.021': 0.134602) '0.105-0.186': 0.037256, (Micropoecilia_picta_Trin: 0.034561, Micropoecilia_picta: 0.034561) '0.017-0.061': 0.147674) '0.142-0.222': 0.028109, ((Micropoecilia_reticulata: 0.004921, Micropoecilia_obscura: 0.004921) '0.000-0.013': 0.036153, (Micropoecilia_wingei_Fel: 0.004979, Micropoecilia_wingei: 0.004979) '0.001-0.011': 0.036095) '0.021-0.067': 0.169269) '0.171-0.256': 0.053863) '0.219-0.312': 0.069100, ((Cnesterodon_septentrionalis: 0.069100, Cnesterodon_decemmaculatus: 0.069100) '0.035-0.131': 0.037561, Cnesterodon_hypselurus: 0.106661) '0.060-0.177': 0.226646) '0.281-0.386': 0.081932) '0.373-0.460': 0.023014, Phalloceros_caudimaculatus: 0.438253) '0.391-0.492': 0.013581, Phalloptychus_januarius: 0.451833) '0.402-0.511': 0.052088, Tomeurus_gracilis: 0.503921) '0.445-0.567': 0.061341, Xenodexia_ctenolepis: 0.565263) '0.501-0.626': 0.103269, (((Jenynsia_multidentata: 0.012565, Jenynsia_lineata: 0.012565) '0.005-0.026': 0.503481, (Anableps_dovii: 0.008406, Anableps_anableps: 0.008406) '0.003-0.017': 0.507640) '0.345-0.632': 0.071796, Oxyzygonectes_dovii: 0.587841) '0.419-0.676': 0.080690) '0.598-0.710': 0.046860, (Fluviphylax_simplex: 0.221188, Fluviphylax_pygmaeus: 0.221188) '0.122-0.385': 0.494203) '0.642-0.771': 0.024594, (((Aplocheilichthys_normani: 0.420762, Aplocheilichthys_spilauchen: 0.420762) '0.275-0.568': 0.171220, Valencia_hispanica: 0.591982) '0.413-0.715': 0.105561, Orestias: 0.697543) '0.598-0.773': 0.042443) '0.666-0.797': 0.041269, (((((((((((((((((Allotoca_diazi: 0.006924, Allotoca_meeki: 0.006924) '0.001-0.016': 0.004922, Allotoca_catarinae: 0.011847) '0.004-0.022': 0.020113, Allotoca_zacapuensis: 0.031960) '0.016-0.052': 0.021071, (Allotoca_dugesii: 0.040718, Allotoca_goslinei: 0.040718) '0.024-0.059': 0.012313) '0.037-0.072': 0.016687, Allotoca_sp_MNCN3676: 0.069718) '0.050-0.095': 0.016866, Allotoca_maculata: 0.086584) '0.062-0.113': 0.028712, Allotoca_regalis: 0.115296) '0.088-0.141': 0.017166, Hubbsina_turneri: 0.132462) '0.107-0.156': 0.007334, ((((Skiffia_francesae: 0.009509, Skiffia_multipunctata: 0.009509) '0.002-0.022': 0.039895, Skiffia_lermae: 0.049404) '0.027-0.077': 0.051874, Skiffia_bilineatus: 0.101278) '0.065-0.134': 0.019438, ((Girardinichthys_multiradiatus: 0.074789, Girardinichthys_viviparus: 0.074789) '0.043-0.111': 0.033316, Neotoca_bilineata: 0.108106) '0.071-0.139': 0.012610) '0.092-0.147': 0.019080) '0.115-0.163': 0.016711, Ataeniobius_toweri: 0.156506) '0.133-0.179': 0.007662, (((((((Chapalichthys_pardalis: 0.010789, Chapalichthys_encaustus: 0.010789) '0.004-0.022': 0.034035, Alloophorus_robustus: 0.044823) '0.028-0.065': 0.005734, Xenotoca_variatus: 0.050558) '0.034-0.070': 0.007702, Ameca_splendens: 0.058260) '0.041-0.078': 0.043547, ((Zoogoneticus_quitzeoensis: 0.064824, Zoogoneticus_tequila: 0.064824) '0.041-0.096': 0.029876, Xenoophorus_captiva: 0.094699) '0.066-0.123': 0.007107) '0.073-0.129': 0.008248, (Xenotoca_melanosoma: 0.056556, Xenotoca_eiseni: 0.056556) '0.029-0.091': 0.053498) '0.081-0.139': 0.030822, (Goodea_gracilis: 0.006255, Goodea_atripinnis: 0.006255) '0.002-0.015': 0.134622) '0.110-0.169': 0.023292) '0.141-0.187': 0.013444, ((((Ilyodon_whitei: 0.004221, Ilyodon_xantusi: 0.004221) '0.001-0.012': 0.014499, (Ilyodon_furcidens: 0.010487, Ilyodon_amecae: 0.010487) '0.002-0.023': 0.008234) '0.008-0.036': 0.075353, Xenotaenia_resolanae: 0.094073) '0.052-0.141': 0.037735, (((Allodontichthys_hubbsi: 0.028606, Allodontichthys_tamazulae: 0.028606) '0.015-0.048': 0.010563, Allodontichthys_polylepis: 0.039169) '0.023-0.061': 0.009019, Allodontichthys_zonistius: 0.048188) '0.029-0.072': 0.083620) '0.089-0.168': 0.045804) '0.155-0.199': 0.011389, (Characodon_lateralis: 0.027054, Characodon_audax: 0.027054) '0.010-0.055': 0.161946) '0.165-0.210': 0.032055, ((Crenichthys_baileyi: 0.049045, Crenichthys_nevadae: 0.049045) '0.028-0.073': 0.021745, Empetrichthys_latos: 0.070789) '0.056-0.095': 0.150267) '0.201-0.230': 0.167457, (Profundulus_punctatus: 0.089375, Profundulus_guatemalensis: 0.089375) '0.039-0.182': 0.299138) '0.286-0.548': 0.073105, Profundulus_labialis: 0.461618) '0.346-0.627': 0.291633, (((Lucania_goodei: 0.018187, Lucania_parvae: 0.018187) '0.006-0.039': 0.354250, (Fundulus_lineolatus: 0.218590, Fundulus_cingulatus: 0.218590) '0.126-0.346': 0.153848) '0.249-0.503': 0.303628, ((Jordanella_floridae: 0.282309, Cyprinodon_variegatus: 0.282309) '0.168-0.432': 0.158492, Floridichthys_carpio: 0.440801) '0.305-0.587': 0.235264) '0.567-0.764': 0.077186) '0.671-0.819': 0.028004) '0.709-0.840': 0.033816, (Cubanichthys_pengelleyi: 0.347044, Cubanichthys_cubensis: 0.347044) '0.177-0.538': 0.468026) '0.743-0.882': 0.092293, (((((Aphyosemion_bitaeniatum: 0.297516, Fundulopanchax: 0.297516) '0.179-0.428': 0.086012, Adamas_formosus: 0.383527) '0.249-0.517': 0.223272, (Aphyoplatys_duboisi: 0.338638, Epiplatys_annulatus: 0.338638) '0.200-0.475': 0.268161) '0.481-0.719': 0.114699, Aplocheilus_lineatus: 0.721498) '0.617-0.812': 0.078528, Rivulus_hartii: 0.800025) '0.706-0.886': 0.107338) '0.845-0.971': 0.053458, (Oryzias_latipes: 0.809365, Atheriniformes: 0.809365) '0.684-0.924': 0.151456) '0.897-1.022': 0.044641, (Oreochromis: 0.507859, Cichlasomatinae: 0.507859) '0.405-0.729': 0.497603) '0.943-1.065': 0.030357, Mugil: 1.035820) '0.975-1.098': 0.064459, (Pleuronectiformes: 0.989679, Monopterus_albus: 0.989679) '0.877-1.077': 0.110600) '1.045-1.160': 0.021524, ((((Tetraodon_nigroviridis: 0.445196, Takifugu_rubripes: 0.445196) '0.340-0.552': 0.540308, Lophius: 0.985504) '0.969-1.027': 0.052923, (Lutjanus: 0.810242, Morone: 0.810242) '0.734-0.841': 0.228185) '1.001-1.092': 0.025837, ((Lycodes: 0.672010, Gasterosteus_aculeatus: 0.672010) '0.456-0.819': 0.231164, Scorpaeniformes: 0.903174) '0.727-1.015': 0.161090) '1.020-1.121': 0.057539) '1.069-1.181': 0.047138, (Ophidiiformes: 0.977370, Porichthys: 0.977370) '0.803-1.091': 0.191570) '1.109-1.237': 0.060562, Beryciformes: 1.229503) '1.165-1.298': 0.070244, Lampriformes: 1.299747) '1.224-1.373': 0.029316, (Polymixia: 1.177246, Zeus_faber: 1.177246) '1.007-1.306': 0.151817) '1.250-1.401': 0.037698, Myctophiformes: 1.366761) '1.287-1.439': 0.025585, Synodus: 1.392346) '1.314-1.463': 0.034831, ((Gadus_morhua: 0.731359, Macrouridae: 0.731359) '0.593-0.925': 0.573649, Aphredoderus_sayanus: 1.305007) '1.165-1.416': 0.122170) '1.348-1.497': 0.141765, (Osmeriformes: 1.384903, Stomiiformes: 1.384903) '1.199-1.519': 0.184039) '1.483-1.620': 0.032541, ((Oncorhynchus_mykiss: 0.929600, Esox_lucius: 0.929600) '0.722-1.167': 0.360513, Argentina: 1.290113) '1.027-1.473': 0.311371) '1.510-1.646': 0.029495, (((((Semotilus_atromaculatus: 0.213016, Notemigonus_crysoleucas: 0.213016) '0.132-0.347': 0.407631, Danio_rerio: 0.620647) '0.415-0.821': 0.668449, ((Characiformes: 0.994509, Ictalurus_punctatus: 0.994509) '0.930-1.107': 0.106260, Gymnotiformes: 1.100768) '1.007-1.221': 0.188328) '1.198-1.386': 0.109459, Chanos_chanos: 1.398554) '1.343-1.479': 0.133429, ((Pellona: 0.846877, Dorosoma_cepedianum: 0.846877) '0.650-1.065': 0.154234, Chirocentrus_dorab: 1.001111) '0.836-1.204': 0.530873) '1.500-1.597': 0.098994) '1.584-1.651': 0.002665;
