# Supplementary material for: The origin and biogeographic diversification of fishes in the family Poeciliidae
Source: PLoS One. 2017 Mar 9;12(3):e0172546. doi: 10.1371/journal.pone.0172546 (PMC5344339; doi:10.1371/journal.pone.0172546)
Supplement: S2 Table — (DOC) [file pone.0172546.s002.doc]

**S2 Table. Area Assignments.**

Areas for 252 extant cyprinodontiform taxa that were included in DEC and DEC + J analyses with BioGeoBears. Absence (0) and presence (1) are indicated for six areas as follows: C = Central America; N = North America; S = South America; W = West Indies; A = Africa; E = Europe + Asia

252 6 (C N S W A E)

Adamas_formosus 000010

Alfaro_cultratus 100000

Alfaro_hubberi 100000

Allodontichthys_hubbsi 100000

Allodontichthys_polylepis 100000

Allodontichthys_tamazulae 100000

Allodontichthys_zonistius 100000

Alloophorus_robustus 100000

Allotoca_catarinae 100000

Allotoca_diazi 100000

Allotoca_dugesii 100000

Allotoca_goslinei 100000

Allotoca_maculata 100000

Allotoca_meeki 100000

Allotoca_regalis 100000

Allotoca_sp_MNCN3676 100000

Allotoca_zacapuensis 100000

Ameca_splendens 100000

Anableps_anableps 001000

Anableps_dowei 100000

Aphyoplatys_duboisi 000010

Aphyosemion_bitaeniatum 000010

Aplocheilichthys_normani 000010

Aplocheilichthys_spilauchen 000010

Aplocheilus_lineatus 000001

Ataeniobius_toweri 100000

Belonesox_belizanus 100000

Brachyrhaphis_cascajalensis 100000

Brachyrhaphis_hartwegi 100000

Brachyrhaphis_holdridgei 100000

Brachyrhaphis_parismina 100000

Brachyrhaphis_rhabdophora 100000

Brachyrhaphis_roseni 100000

Brachyrhaphis_terrabensis 100000

Carlhubbsia_kidderi 100000

Carlhubbsia_stuarti 100000

Chapalichthys_encaustus 100000

Chapalichthys_pardalis 100000

Characodon_audax 100000

Characodon_lateralis 100000

Cnesterodon_decemmaculatus 001000

Cnesterodon_hypselurus 001000

Cnesterodon_septentrionalis 001000

Crenichthys_baileyi 010000

Crenichthys_nevadae 010000

Cubanichthys_cubensis 000100

Cubanichthys_pengelleyi 000100

Cyprinodon_variegatus 010000

Girardinus_denticulatus 000100

Girardinus_ramsdeni 000100

Girardinus_sp_DDEN10 000100

Empetrichthys_latos 010000

Epiplatys_annulatus 000010

Floridichthys_carpio 010000

Fluviphylax_pygmaeus 001000

Fluviphylax_simplex 001000

Fundulopanchax 000010

Fundulus_cingulatus 010000

Fundulus_lineolatus 010000

Gambusia_affinis 010000

Gambusia_atrora 010000

Gambusia_caymanensis 000100

Gambusia_eurystoma 100000

Gambusia_geiseri 010000

Gambusia_heterochir 010000

Gambusia_hispaniolae 000100

Gambusia_holbrooki 010000

Gambusia_hubbsi 000100

Gambusia_hurtadoi 010000

Gambusia_luma 100000

Gambusia_manni 000100

Gambusia_marshi 010000

Gambusia_melapleura 000100

Gambusia_nicaraguensis 100000

Gambusia_oligosticta 000100

Gambusia_panuco 100000

Gambusia_punctata 000100

Gambusia_puncticulata 000100

Gambusia_rhizophorae 010100

Gambusia_sexradiata 100000

Gambusia_sp_LLSTC4571 100000

Gambusia_vittata 100000

Gambusia_wrayi 000100

Gambusia_yucatana 100000

Gambusia_zarskei 100000

Girardinichthys_multiradiatus 100000

Girardinichthys_viviparus 100000

Girardinus_creolus 000100

Girardinus_metallicus 000100

Girardinus_microdactylus 000100

Girardinus_rivasi 000100

Girardinus_sp_GMIC19 000100

Girardinus_falcatus 000100

Girardinus_uninotatus 000100

Goodea_atripinnis 100000

Goodea_gracilis 100000

Heterandria_bimaculata 100000

Heterandria_formosa 010000

Heterandria_jonesi 100000

Heterophallus_milleri 100000

Heterophallus_rachovii 100000

Hubbsina_turneri 100000

Ilyodon_amecae 100000

Ilyodon_furcidens 100000

Ilyodon_whitei 100000

Ilyodon_xantusi 100000

Jenynsia_lineata 001000

Jenynsia_multidentata 001000

Jordanella_floridae 010000

Limia_caymanensis 000100

Limia_dominicensis 000100

Limia_garnieri 000100

Limia_grossidens 000100

Limia_heterandria 001000

Limia_melanogaster 000100

Limia_melanonotata 000100

Limia_nigrofasciata 000100

Limia_pauciradiata 000100

Limia_perugiae 000100

Limia_rivasi 000100

Limia_sulfurophila 000100

Limia_tridens 000100

Limia_versicolor 000100

Limia_vittata 000100

Limia_zonata 000100

Lucania_goodei 010000

Lucania_parvae 010000

Micropoecilia_bifurca 001000

Micropoecilia_minima 001000

Micropoecilia_obscura 001000

Micropoecilia_parae_French_Guiana 001000

Micropoecilia_parae_Suriname 001000

Micropoecilia_picta 001000

Micropoecilia_picta_Trin 001000

Micropoecilia_reticulata 001000

Micropoecilia_sarrafae 001000

Micropoecilia_wingei 001000

Micropoecilia_wingei_Venezuela 001000

Neoheterandria_cana 100000

Neoheterandria_elegans 001000

Neoheterandria_tridentiger 100000

Neotoca_bilineata 100000

Orestias 001000

Oxyzygonectes_dovii 100000

Pamphorichthys_araguaiensis 001000

Pamphorichthys_hasemani 001000

Pamphorichthys_hollandi 001000

Pamphorichthys_minor 001000

Pamphorichthys_scalpridens 001000

Phallichthys_amates 100000

Phallichthys_pittieri 100000

Phallichthys_quadripunctatus 100000

Phallichthys_tico 100000

Phalloceros_caudimaculatus 001000

Phalloptychus_januarius 001000

Poecilia_butleri_MR04 100000

Poecilia_catemaconis 100000

Poecilia_caucana 100000

Poecilia_chica 100000

Poecilia_gilli 100000

Poecilia_gracilis 100000

Poecilia_latipinna 010000

Poecilia_latipunctata 100000

Poecilia_mexicana_lim 100000

Poecilia_mexicana_mex 100000

Poecilia_orri 100000

Poecilia_petenensis_Campeche 100000

Poecilia_petenensis_MP523 100000

Poecilia_salvatoris 100000

Poecilia_sphenops 100000

Poecilia_sulphuraria 100000

Poecilia_velifera_MP737 100000

Poecilia_vivipara 001100

Poecilia_vivipara_Trinidad 001000

Poeciliopsis_baenschi 100000

Poeciliopsis_balsas 100000

Poeciliopsis_catemaco 100000

Poeciliopsis_elongata 100000

Poeciliopsis_fasciata 100000

Poeciliopsis_gracilis 100000

Poeciliopsis_hnilickai 100000

Poeciliopsis_infans 100000

Poeciliopsis_latidens 100000

Poeciliopsis_lucida 100000

Poeciliopsis_monacha 100000

Poeciliopsis_occidentalis 010000

Poeciliopsis_paucimaculata 100000

Poeciliopsis_pleurospilus 100000

Poeciliopsis_presidionis 100000

Poeciliopsis_prolifica 100000

Poeciliopsis_retropinna 100000

Poeciliopsis_scarlii 100000

Poeciliopsis_sonoriensis 010000

Poeciliopsis_turneri 100000

Poeciliopsis_turrubarensis 100000

Poeciliopsis_viriosa 100000

Priapella_chamulae 100000

Priapella_compressa 100000

Priapella_intermedia 100000

Priapella_olmecae 100000

Priapichthys_annectens 100000

Priapichthys_darienensis 100000

Pseudopoecilia_festae 001000

Priapichthys_panamensis 100000

Priapichthys_puetzi 100000

Profundulus_guatemalensis 100000

Profundulus_labialis 100000

Profundulus_punctatus 100000

Quintana_atrizona 000100

Rivulus_hartii 001000

Scolichthys_greenwayi 100000

Scolichthys_iota 100000

Skiffia_bilineatus 100000

Skiffia_francesae 100000

Skiffia_lermae 100000

Skiffia_multipunctata 100000

Tomeurus_gracilis 001000

Valencia_hispanica 000001

Xenodexia_ctenolepis 100000

Xenoophorus_captiva 100000

Xenophallus_umbratilis 100000

Xenotaenia_resolanae 100000

Xenotoca_eiseni 100000

Xenotoca_melanosoma 100000

Xenotoca_variatus 100000

Xiphophorus_alvarezi 100000

Xiphophorus_andersi 100000

Xiphophorus_birchmanni 100000

Xiphophorus_clemenciae 100000

Xiphophorus_continens 100000

Xiphophorus_cortezi 100000

Xiphophorus_couchianus 100000

Xiphophorus_evelynae 100000

Xiphophorus_gordoni 100000

Xiphophorus_hellerii 100000

Xiphophorus_maculatus 100000

Xiphophorus_malinche 100000

Xiphophorus_mayae 100000

Xiphophorus_meyeri 100000

Xiphophorus_milleri 100000

Xiphophorus_mixei 100000

Xiphophorus_montezumae 100000

Xiphophorus_monticolus 100000

Xiphophorus_multilineatus 100000

Xiphophorus_nezahualcoyotl 100000

Xiphophorus_nigrensis 100000

Xiphophorus_pygmaeus 100000

Xiphophorus_signum 100000

Xiphophorus_variatus 100000

Xiphophorus_xiphidium 100000

Zoogoneticus_quitzeoensis 100000

Zoogoneticus_tequila 100000
