# Supplementary material for: The origin and biogeographic diversification of fishes in the family Poeciliidae
Source: PLoS One. 2017 Mar 9;12(3):e0172546. doi: 10.1371/journal.pone.0172546 (PMC5344339; doi:10.1371/journal.pone.0172546)
Supplement: S3 Table — (DOCX) [file pone.0172546.s003.docx]

S3 Table. Details of colonization events summarized in Table 4.

Estimated dates of dispersal of a species or common ancestor of a clade to a new area. Here we detail the sixteen dispersal events summarized in Table 4. MRCA = most recent common ancestor.

| Colonization Event | Autocorrelated/Independent* |
| --- | --- |
| *Xenodexia* from SA to CA | < 53.4 Ma, < 56.5 Ma |
| ^#^MRCA of *Gambusia* + *Poeciliopsis* from SA to CA | 40.8 – 37.2 Ma, 41.5 – 37.5 Ma |
| ^#^MRCA of *Girardinus* + *Quintana* from SA to Cuba | 40.8 – 25.9 Ma, 41.5 – 25.5 Ma |
| MRCA of *Mollienesia* from SA to CA/Mexico | 25.5 – 19.0 Ma, 20.1 – 14.3 Ma |
| *Mollienesia latipinna* from CA/Mexico to NA | < 6.3 Ma, < 3.6 Ma |
| *Pseudopoecilia festae* from CA to SA | < 24.4 Ma, < 22.5 Ma |
| *Neoheterandria elegans* from CA to SA | < 21.4 Ma, < 21.7 Ma |
| *Heterandria formosa* from CA to NA | < 25.7 Ma, < 22.8 Ma |
| MRCA of *Limia* (excluding *L. heterandria*) from SA to Caribbean | 22.8 – 16.2 Ma, 16.7 – 11.4 Ma |
| MRCA of *Gambusia atrora*, *G. marshi*, *G. hurtadoi*, and *G. rhizophorae* from CA to NA | 9.3 – 8.7 Ma, 11.4 – 10.4 Ma |
| MRCA of *Gambusia affinis* and *G. geiseri* from CA to NA | 11.0 – 9.7 Ma, 14.1 – 11.6 Ma |
| *Gambusia vittata* from NA to Mexico | < 4.4 Ma, < 4.4 Ma |
| *Gambusia panuco* from NA to Mexico | < 4.4 Ma, < 4.1 Ma |
| MRCA of *Gambusia rhizophorae* and *G. punctata* from NA to West Indies | 8.7 – 4.4 Ma, 10.4 – 5.1 Ma |
| MCRA of *Gambusia puncticulata*, *G. caymanensis*, and *G. oligosticta* from CA/Mexico to West Indies | 3.4 – 1.1 Ma, 4.7 – 2.1 Ma |
| MRCA of *Gambusia manni* and *G. hubbsi* from CA/Mexico to West Indies | 5.5 – 1.2 Ma, 6.0 – 1.6 Ma |
| MRCA of *Gambusia melapleura*, *G. wrayi*, and *G. hispaniolae* from CA/Mexico to West Indies | 10.5 – 6.4 Ma, 13.3– 7.7 Ma |
| *Gambusia* sp. LLSTC4571 from NA to CA/Mexico | < 1.6 Ma, < 2.6 Ma |

*Point estimates based on mcmctree analyses with autocorrelated and independent rates, respectively.

^#^Reconstruction of colonization event is equivocal and spans two internal nodes.

Abbreviations: CA = Central America; NA = North America; SA = South America.
